# Enhancing Antibacterial Activity of *Medinilla speciosa* Blume Fruits Against *Cutibacterium acnes* Through Phytosome Delivery: An In Vivo Study

**DOI:** 10.3390/ph19060825

**Published:** 2026-05-25

**Authors:** Ririn Puspadewi, Tiana Milanda, Muhaimin Muhaimin, Anis Yohana Chaerunisaa, Sri Agung Fitri Kusuma, Yuni Elsa Hadisaputri, Faizal Hermanto, Lia Mardiana

**Affiliations:** 1 Doctoral Program in Pharmacy, Faculty of Pharmacy, Universitas Padjadjaran, Sumedang 45363, Indonesia; ririn22003@mail.unpad.ac.id (R.P.); lia22006@mail.unpad.ac.id (L.M.); 2 Faculty of Pharmacy, Universitas Jenderal Achmad Yani, Cimahi 40531, Indonesia; faizal.hermanto@lecture.unjani.ac.id; 3 Faculty of Pharmacy, Universitas Padjadjaran, Sumedang 45363, Indonesia; muhaimin@unpad.ac.id (M.M.); anis.yohana.chaerunisaa@unpad.ac.id (A.Y.C.); s.a.f.kusuma@unpad.ac.id (S.A.F.K.); yuni.elsa@unpad.ac.id (Y.E.H.); 4 Center for Herbal Studies, Universitas Padjadjaran, Sumedang 45363, Indonesia; 5 Faculty of Pharmacy, Islamic University of Kalimantan Muhammad Arsyad Al-Banjari, Banjarmasin 70123, Indonesia

**Keywords:** antibacterial, *C. acnes*, phytosome, inflammation, acne, *M. speciosa*

## Abstract

**Background/Objectives**: The fruit of *Medinilla speciosa* Blume fruit contains flavonoids with potent activity against *Cutibacterium acnes*, but their clinical application is hindered by poor bioavailability. This study aimed to develop, characterize, and evaluate a phytosome-based vesicular system to enhance the in vivo antibacterial efficacy of the fruit’s ethyl acetate fraction (EAFMS). **Methods**: Phytosomes were synthesized via antisolvent precipitation using a 1:3 EAFMS-to-phospholipid ratio. Formulations were characterized for particle size, polydispersity index (PDI), zeta potential, entrapment efficiency (EE), and in vitro release. Antibacterial efficacy was assessed in *C. acnes*-induced Wistar rats over three days. **Results**: EAFMS showed superior antibacterial activity with a 93.5% relative potency compared to tetracycline. The optimized phytosomes exhibited favorable physicochemical properties: particle size of phytosome 244.60 ± 0.85 nm, PDI of phytosome 0.396 ± 0.08, zeta potensial of phytosome −56.70 ± 2.08 mV, and EE of phytosome 89.46 ± 0.45%. The formulation achieved a 76.504% cumulative release at 8 h. In vivo trials demonstrated that the phytosome cream significantly reduced bacterial colony counts and diminished inflammatory cell infiltration compared to the cream base. **Conclusions**: The phytosome system effectively improves the stability and delivery of *M. speciosa* flavonoids, significantly enhancing their antibacterial and anti-inflammatory performance against acne.

## 1. Introduction

Acne is a chronic inflammation of the sebaceous follicles that is triggered by various factors, especially *Cutibacterium acnes* bacterial infection. These bacteria trigger the release of various pro-inflammatory cytokines such as IL-1*β*, IL-6, IL-8, and TNF-*α* through the activation of keratinocyte, monocyte, and sebocyte cells. Although synthetic antibiotics are effective for treatment, long-term use risks leading to bacterial resistance and skin irritation, so the exploration of natural ingredients becomes crucial [1,2].

One of the promising natural ingredients is parijoto fruit (*Medinilla speciosa* Blume) which is rich in phenolic compounds and flavonoids with antibacterial and anti-inflammatory activity. However, the flavonoid compounds of this plant are hydrophilic with a large molecular size, making it difficult to penetrate the lipid layer of the skin. This limitation of lipid solubility leads to poor absorption and low bioavailability at target skin cells [3,4]. Notably, flavonoids (the active compounds found in this plant) possess hydrophilic properties. These compounds typically have large molecular sizes and poor lipid solubility [5,6]. Because human cell membranes, including the intestinal wall and skin layers, consist primarily of lipid structures, these active plant compounds face significant challenges in penetrating target cells. Consequently, absorption within the body decreases, leading to low bioavailability [7,8].

Phytosome technology has been developed to address challenges related to the inherent properties of plant phytoconstituents. These delivery systems consist of lipids and bioactive compounds, which facilitate enhanced solubility and bioavailability. Phospholipids, most commonly phosphatidylcholine, serve as the lipid phase substances utilized to create compatible lipid phytoconstituents. Over recent years, phytosome technology has achieved significant progress across the pharmaceutical, cosmetic, and nutraceutical sectors, supporting formulations in various forms such as powders, granules, tablets, gels, emulsions, lotions, solutions, and capsules. Distinctively, phytosomes exhibit a chemical bond between the phospholipid molecules and the phytoconstituents, thereby ensuring greater structural stability. Beyond their role as carriers, phosphatidylcholine molecules also possess inherent therapeutic properties that provide a synergistic effect when utilized in phytosome formation. The phospholipid molecule is vital because it consists of a water-soluble head and two fat-soluble tails. Consequently, these molecules achieve dual solubility and function as highly effective emulsifiers [9,10].

Furthermore, phospholipid molecules act as a potential vehicle system to improve the bioavailability of extracts or phytoconstituents that are typically poorly absorbed. Due to their unique structural components, which are equivalent to the lipid content of mammalian cell membranes, these complexes are highly compatible with physiological systems [11,12]. Phytosomes were specifically selected for their high flavonoid content. Unlike Solid Lipid Nanoparticles (SLNs), which may experience drug leaching during storage, the hydrogen bonds between flavonoids and phosphatidylcholine in phytosomes protect the polyphenol ring from oxidative degradation and premature metabolism, ensuring that its pharmacological antioxidant activity reaches the skin layers intact [10].

In this current study, an ethyl acetate fraction of *Medinilla speciosa* Blume (EAFMS) was formulated into phytosomes to improve bioavailability and enhance therapeutic efficacy. These phytosomes are produced through a process where plant extracts or bioactives bind to phospholipids, primarily phosphatidylcholine, resulting in a lipid-compatible molecular complex. In this case, water-soluble phytoconstituents are transformed into these specialized lipid-compatible molecular complexes [13].

To date, the antibacterial effects of *M. speciosa* Blume fruit in phytosomal form have remained unexplored. When applied directly to the skin as a standard extract, hydrophilic phytoconstituents tend to be trapped within the outermost skin layer, the stratum corneum, or repelled by sebum inside the pores. Consequently, these antibacterial compounds fail to reach the deep-seated locations where *C. acnes* bacteria aggregate [14]. There is therefore a clear need for research to determine whether formulating *M. speciosa* Blume fruit into phytosomes can successfully enhance its inhibitory activity against *C. acnes* growth. Phytosome preparation was executed using a ratio of 1:3 of ethyl acetate fraction of *M. speciosa* Blume (EAFMS) to soy lecithin. These phytosomes were characterized based on particle size, polydispersity index, zeta potential, and entrapment efficiency. To evaluate long-term stability, samples were stored at temperatures of 25 °C and 40 °C for periods of 30, 60, and 90 days before undergoing re-characterization. Furthermore, antibacterial activity testing against *C. acnes* was conducted in vivo using animal subjects induced with the bacteria, alongside an assessment of the in vitro release profile.

## 2. Results

### 2.1. Antibacterial Activity of Extracts and Fractions Against C. acnes

The collected data reveal a consistent pattern of increasing inhibition zone diameters for the extract and all subsequent fractions (see Table 1). Correspondingly, the ethyl acetate fraction exhibited the most substantial inhibitory effect across all tested concentrations. The *n*-hexane and water fractions demonstrated activity levels below those of the ethyl acetate fraction. Specifically, the percentage of activity for the ethanol extract relative to tetracycline reached 82.35%, while the ethyl acetate fraction reached 93.52%. In contrast, the *n*-hexane and water fractions showed relative activities of 68.61% and 76.79%, respectively. Although the ethyl acetate fraction achieved a high potency of 93.5%, it is important to note that the concentration required (50 mg/mL) was significantly higher than that of tetracycline (0.03 mg/mL).

### 2.2. Phytosome Preparation and Optimization

Following the results of the antibacterial activity assays, phytosomes were formulated using the ethanol extract and the ethyl acetate fraction. The EAFMS-phytosome complexes were synthesized via the antisolvent precipitation method. This specific technique is frequently favored in phytosome development due to its overall effectiveness. Essentially, the process involves dissolving the phospholipid-active ingredient complex in an organic solvent, which is then introduced into a non-polar solvent (antisolvent) to trigger precipitation [10]. The resulting formulations were subsequently evaluated for particle size and zeta potential (see Table 2).

Based on the characterization results obtained for the extract and various fractions, the ethyl acetate fraction phytosome was selected for further stability testing and in vivo antibacterial evaluation against *C. acnes*.

### 2.3. Phytosome Characterization

The results for phytosome characterization indicate that the phytosomes (see Table 3) remained stable throughout 90 days of storage at 25 °C and 40 °C. Statistically, neither temperature variations nor the duration of storage exerted a significant influence on the entrapment efficiency, as evidenced by a significance value of 0.661 (*p* > 0.05).

The initial particle size of this formulation is 244.60 nm. After 90 days, the particle size at 25 °C increased slightly to 252.70 nm. At the accelerated temperature test (40 °C) on day 90, the particle size reached 256.13 nm. PDI on day 0 was recorded at 0.396. Over the course of 90 days, the PDI value fluctuated in the range of 0.417 to 0.529 at 25 °C, and 0.371 to 0.53 at 40 °C. The initial zeta potential is negative, which is −56.70 mV. This value decreased slightly (to be less negative) on the 90th day, namely −53.36 mV at 25 °C and −53.90 mV at 40 °C. The entrapment efficiency on day 0 was very high, namely 89.12%. This figure was maintained remarkably consistently until the 90th day, measured at 89.39% at 25 °C and 89.39% at 40 °C.

### 2.4. Surface Morphology of Phytosomes

**Figure 1:**
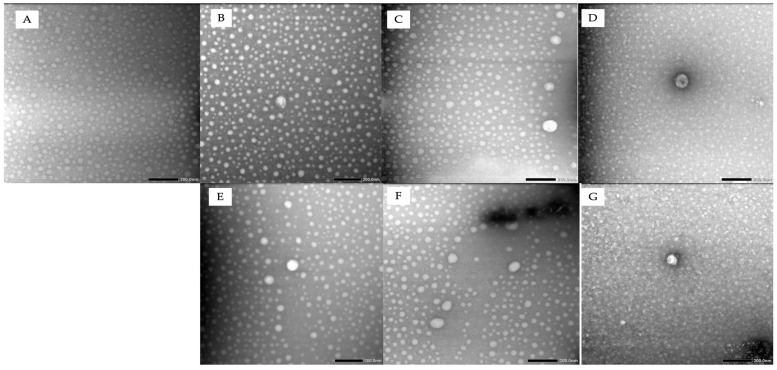
Observation of phytosomes after storage at 25 °C: (**A**) day 0, (**B**) 30 days, (**C**) 60 days, (**D**) 90 days; and at 40 °C: (**E**) 30 days, (**F**) 60 days, (**G**) 90 days.

The surface morphology of the phytosomal fraction was analyzed using transmission electron microscopy (TEM Jeol JEM-1400, Tokyo, Japan). As illustrated in Figure 1, the TEM images reveal that the *M. speciosa* phytosome fraction possesses a spherical surface [15].

### 2.5. Results of FTIR Characterization

**Figure 2:**
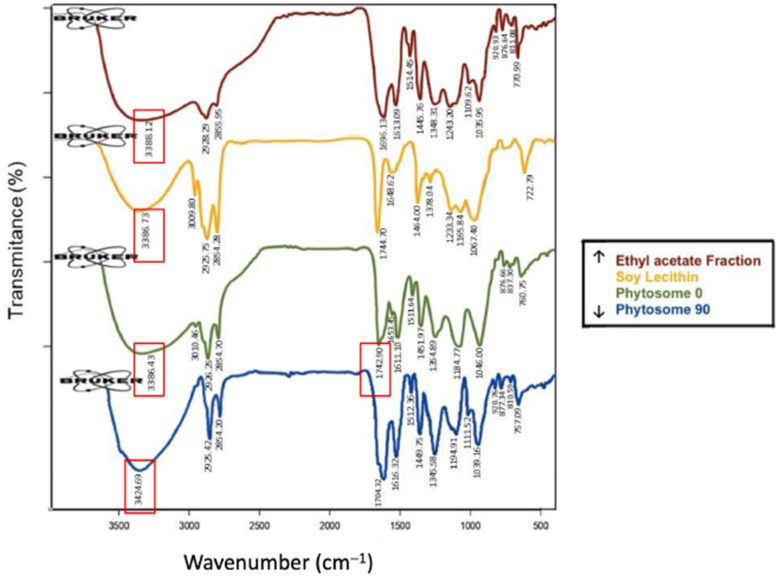
FTIR spectra of ethyl acetate fraction of *M. speciosa* (EAFMS), soy lecithin, phytosome complex at day 0, and phytosome complex at day 90. The significant broadening and shifting of the hydroxyl (–OH) absorption bands in the 3300–3500 cm^−1^ region, along with the shifts in the carbonyl (C=O) stretching vibrations at 1742.90 cm^−1^, indicate the formation of intermolecular hydrogen bonds between the EAFMS and the phospholipid matrix. The spectral consistency between phytosome 0 and phytosome 90 confirms the chemical stability and structural integrity of the complex throughout the 90-day storage period.

FTIR results for the ethyl acetate fraction show O-H group stretching detected at 3388.12 cm^−1^ with high intensity. Additionally, stretching of the C=O aryl ketone group appears at 1669.13 cm^−1^ with strong intensity. Aromatic ring C=C stretching bands are detected at 1613.09, 1514.45, and 1445.76 cm^−1^. The band at 1243.20 cm^−1^ is attributed to C-O stretching in the aryl ether ring, while aromatic C-H stretching emerges at 770.99 cm^−1^ with weak intensity (see Figure 2) [16].

FTIR analysis of the soy lecithin and ethyl acetate fraction complex indicates several absorption peaks across various regions (see Figure 2). This analysis was conducted specifically to identify molecular interactions between the soy lecithin and the EAFMS. Notably, no new peaks emerged within the physical phytosome mixture. Small shifts in the stretching vibration peaks related to −P=O (1183.18 and 1187.79 cm^−1^) in the phosphate group of the phospholipid molecule were observed in the phytosome spectra after 30 days of storage at 25 °C and 40 °C. Shifts also occurred in the −C=O carbonyl ketone group (1617.41 cm^−1^). Furthermore, the sharp peak associated with free O-H stretching vibrations (3406.94 cm^−1^) shifted during the initial preparation and through 30 days of storage. These findings strongly suggest the formation of hydrogen bonds between the free O-H groups in the EAFMS and the phospholipids. Accordingly, it can be concluded that no new conjugative bonds were formed between the ethyl acetate fraction and the phospholipids. Instead, weak intermolecular interactions, such as hydrogen bonding, likely exist between the hydroxyl groups of the ethyl acetate fraction and the carbonyl and phosphoryl groups of the phospholipids. These FTIR results align closely with those reported in previous studies [17,18,19].

### 2.6. Differential Scanning Calorimetry

**Figure 3:**
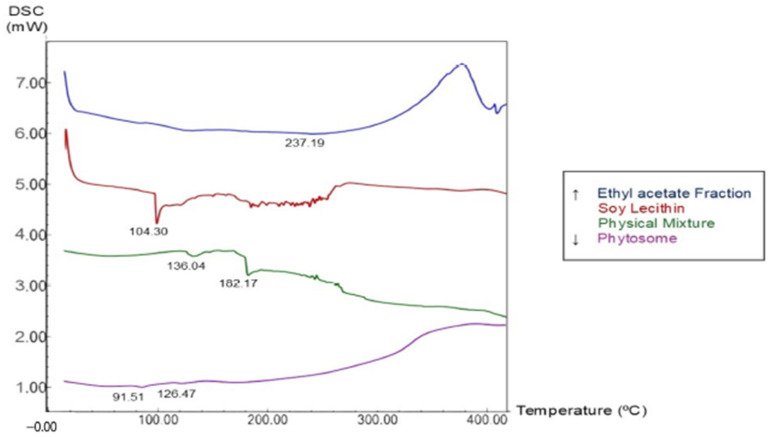
DSC thermogram of ethyl acetate fraction, soy lecithin, physical mixture, and phytosome.

The thermogram of the ethyl acetate fraction (Figure 3) exhibits a relatively flat profile with a broad endothermic peak detected at approximately 237.19 °C. This broadened profile, rather than a sharp peak, indicates that the fraction is a multicomponent mixture of various phytoconstituents with diverse thermal characteristics, further suggesting a predominantly semi-crystalline or amorphous matrix.

The soy lecithin thermogram shows a sharp endothermic peak at 104.30 °C, which is characteristic of pure phospholipids. This peak represents the gel-to-liquid crystalline phase transition temperature or is associated with the melting of the polar head group (choline). Beyond 200 °C, fluctuations in the curve denote the beginning of thermal degradation or oxidation in the fatty acid chains.

The physical mixture’s thermogram shows a clear change in its thermal profile. The sharp endothermic peak of soy lecithin at 104.30 °C shifted, appearing as new peaks at 136.04 °C and 182.17 °C. The phytosome thermogram showed a marked change compared to the curves of the raw materials and the physical mixture, characterized by the disappearance of the endothermic peaks of soy lecithin at 104.30 °C and the fraction at 237.19 °C.

### 2.7. In Vitro Skin Permeation Study

**Figure 4:**
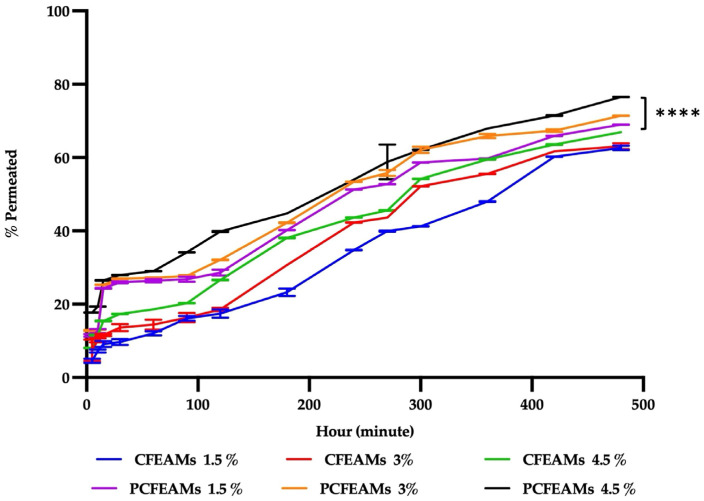
In vitro permeation profile of total flavonoids from CFEAMS and PCFEAMS. Data are presented as mean ± SD (*n* = 3). Statistical analysis was performed using Two-Way ANOVA, showing significant differences based on formulation type (*p* = 0.0001). Tukey’s post hoc test confirms that all PCFEAMS formulations significantly outperformed their corresponding CFEAMS counterparts (*p* < 0.05), **** *p* < 0.0001.

The penetration of cream comprising 1.5%, 3%, and 4.5% of the ethyl acetate fraction of *M. speciosa* fruits (CFEAMS) and the phytosome ethyl acetate fraction (PCFEAMS) through rat skin membranes over 8 h yielded values of 62.65%, 63.11%, 66.88%, 71.39% and 76.50% respectively (see Figure 4). These data demonstrate that the phytosome formulations achieve a higher penetration percentage (68.98% to 76.50%) compared to the fractions without phytosomes (62.65% to 66.88%). Consequently, statistical analysis confirms that the type of cream formulation (phytosome cream versus fraction cream) and the specific concentration significantly influence the in vitro release values, as indicated by a significance level of 0.0001 (*p* < 0.05).

### 2.8. In Vivo Antibacterial Activity of Phytosome Against C. acnes

#### 2.8.1. Bacterial Colony Quantification

**Figure 5:**
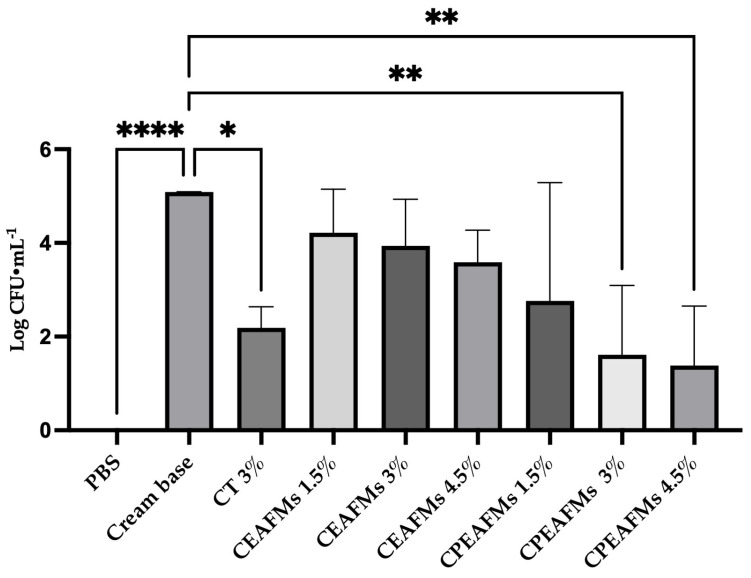
In vivo antimicrobial activity of PCFEAMS at 1.5%, 3%, and 4.5% against *C. acnes*. The bacterial load in the skin tissue was quantified as log CFU·mL^−1^. Data represent the mean ± SD from triplicate (*n* = 3) experiments. Statistical significance was determined by One-Way ANOVA (*p* < 0.05) followed by Dunnet’s multiple comparisons test. Asterisks indicate significant differences compared to the basis group * (*p* < 0.05), ** (*p* < 0.01), **** (*p* < 0.0001).

The bacterial count results depicted in Figure 5 highlight a significant reduction in the quantity of *C. acnes* following the administration of PCFEAMS. Conversely, the negative control group (treated with the cream base) showed a much higher bacterial load. No bacteria were detected in the control group of rats injected only with PBS, which confirms the absence of bacterial contamination during the trial. This evidence effectively demonstrates that PCFEAMS can kill *C. acnes* within a physiological environment like the skin.

#### 2.8.2. Histology and Immunohistochemistry

**Figure 6:**
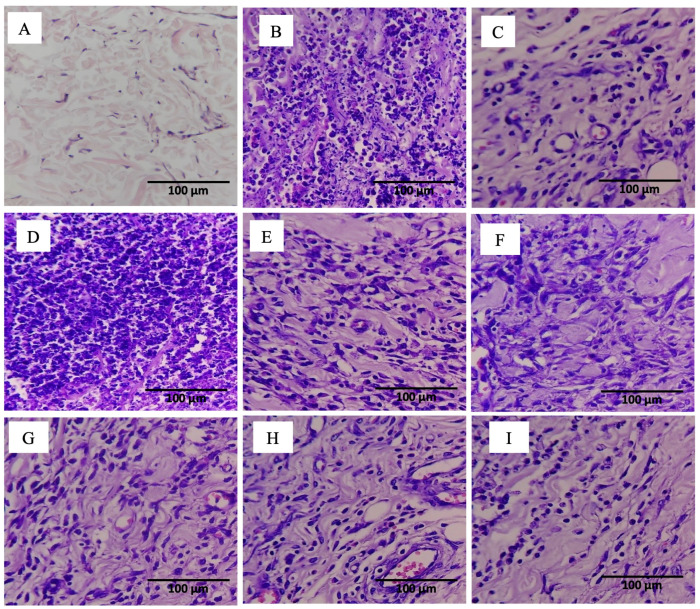
Hematoxylin and eosin staining of experimental groups treated with (**A**) PBS, (**B**) cream base (negative control), (**C**) 3% tetracycline cream (positive control), different concentrations of CFEAMS at (**D**) 1.5%, (**E**) 3%, and (**F**) 4.5%, and PCFEAMS at concentrations of (**G**) 1.5%, (**H**) 3%, and (**I**) 4.5%, at 400× magnification.

The results of hematoxylin and eosin (H&E) staining for each treatment group are presented in Figure 6. Notably, the group administered PBS shows connective tissue with scattered cells and a dominant pink (eosinophilic) extracellular matrix. The visible cells possess small, slender nuclei stained bluish-purple. Furthermore, there is no significant evidence of neutrophil infiltration. Neutrophils typically appear as small cells with multilobed (segmented) nuclei that are intensely basophilic (dark blue). Consequently, this group exhibits minimal to no signs of acute inflammation. In contrast, the group treated with the cream base displays a higher cellular density compared to the PBS group. Within this sample, numerous cells with dense, dark purple nuclei are visible, and several cells appear to have segmented or multilobed nuclei. The extracellular matrix between these cells also seems more edematous or disrupted. Additionally, many small cells with dark, segmented nuclei characteristic of neutrophils are evident. The density of these inflammatory cells is quite striking. Such a high concentration of inflammatory cells, including neutrophils, serves as a strong indicator of an active inflammatory process. Regarding the group treated with 3% tetracycline cream, the images show an increase in cellularity compared to the PBS group, yet the density is not as high as that observed in the cream base group. Cells with dark purple nuclei appear scattered among the tissue fibers. Accordingly, neutrophil infiltration is not as extensive when compared to the negative control. The group treated with 1.5% CFEAMS shows many cells with distinct purple nuclei, indicating an ongoing inflammatory condition. Similarly, the 3% and 4.5% CFEAMS groups display purple-stained cells, but their distribution is less dense than that of the 1.5% concentration. Most significantly, the groups administered PCFEAMS across all concentrations show areas dominated by pink (eosinophilic) staining, with inflammatory markers being vastly reduced compared to the CFEAMS groups.

**Figure 7:**
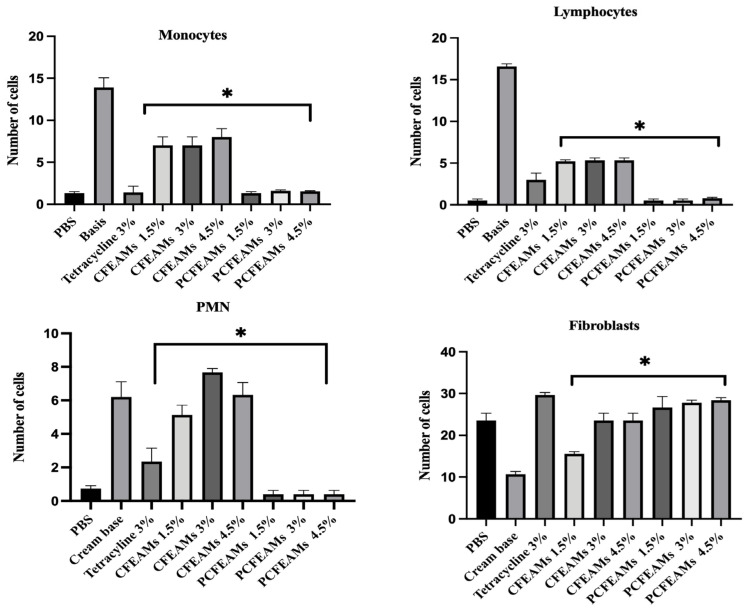
Histological quantification of inflammatory cells and fibroblast. The bar chart represents the number of monocytes, lymphocytes, PMN, and fibroblast. Data are expressed as mean ± SD from triplicate experiments (*n* = 3). Statistical significance was determined by One-Way ANOVA followed by Tukey’s post hoc test for multiple comparisons. The asterisk (*) denotes a significant difference (*p* < 0.05).

The data presented in Figure 7 reveal that the base group exhibits a high count of inflammatory cells, with monocyte levels at 13.96, lymphocytes at 16.60, and PMN at 6.20. These figures are significantly higher than those observed in the group receiving only PBS without induction. Evidently, the injection of *C. acnes* triggers a substantial infiltration into the dermis. Furthermore, the elevated monocyte count in the base group suggests that *C. acnes* cells activate monocytes through the TLR2/NF-*κβ* pathway. This activation subsequently triggers the production of pro-inflammatory cytokines such as TNF-*α*, IL-1*β*, IL-6, and IL-8. Across the three PCFEAMS concentration groups (1.5%, 3.0%, and 4.5%), the mean monocyte/lymphocyte counts were 1.45 ± 0.21, 1.38 ± 0.18, and 1.33 ± 0.15, respectively. One-Way ANOVA revealed a statistically significant difference among treatment groups. Tukey’s HSD post hoc test indicated that all three PCFEAMS groups differed significantly from the cream base control (*p* < 0.05), while no significant difference was observed between the 3.0% and 4.5% PCFEAMS groups (*p* < 0.05). These data suggest a concentration-dependent trend in the reduction in inflammatory cell infiltration, although the effect appears to plateau at the 3.0% concentration.

A similar pattern emerged regarding lymphocyte cells. The group treated with the base alone showed a high lymphocyte count of 16.60, whereas lower values were observed in the ethyl acetate fraction (5.20 to 5.33), tetracycline (3.00), and phytosome (0.50 to 0.80) groups. The significant reduction of lymphocytes by PCFEAMS demonstrates strong inhibition of the immune components involved in acne inflammation.

Finally, the PMN cell count in the base group reached 6.20, while the PCFEAMS groups maintained much lower levels at 0.40. Tetracycline resulted in a PMN count of 2.33, aligning with its known anti-inflammatory properties. Tetracycline significantly decreases the production of major pro-inflammatory cytokines such as TNF-*α*. This reduction is specifically linked to the inhibition of the NF-*κβ* pathway, which plays a central role in regulating the gene expression of inflammatory cytokines and chemokines. Findings from specific signaling pathway inhibitors reinforce the conclusion that NF-*κβ* is the primary pathway modulated by tetracycline to suppress cytokine production [20].

Fibroblast data indicate that the PBS group reached a value of 23.53, which serves as the baseline for fibroblast activity without any specific treatment. Conversely, the base group showed a lower value of 10.66, signifying a slight decline in fibroblast activity. Tetracycline exhibited a higher value of 29.66, demonstrating an increase in fibroblast activity. Regarding the CFEAMS groups, the recorded values (1.5% = 15.53; 3% = 23.53; 4.5% = 23.53) were lower than those of the 3% tetracycline and remained close to the base group results. More effectively, the phytosome formulations (1.5% = 26.66; 3% = 27.80; 4.5% = 28.40) showed fibroblast values approaching those achieved by the 3% tetracycline treatment.

**Figure 8:**
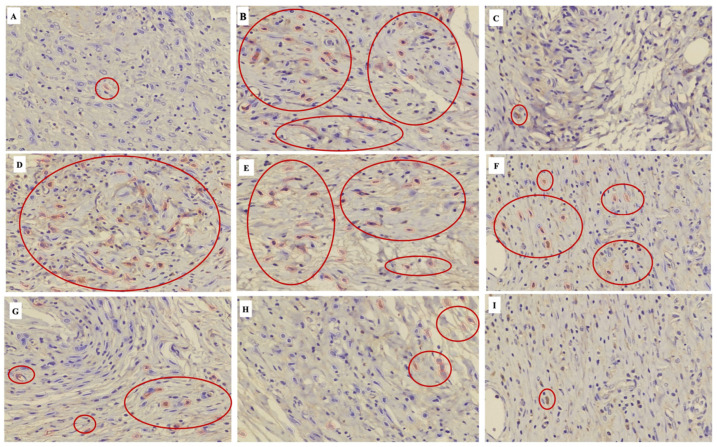
Immunohistochemical imagery of TNF-*α* expression in experimental groups treated with (**A**) PBS, (**B**) cream base (negative control), (**C**) 3% tetracycline cream (positive control), different concentrations of CFEAMs: (**D**) 1.5%, (**E**) 3%, and (**F**) 4.5%, and PCFEAMs at concentrations: (**G**) 1.5%, (**H**) 3%, and (**I**) 4.5%, at 400× magnification.

Immunohistochemistry results (see Figure 8) for the experimental animals receiving the cream base as a negative control reveal brown staining in several cells. The presence of these brown stains indicates the specific sites (marked with a red circle) where the TNF-*α* antibody has successfully bound. When viewed as a whole, this group displays the most extensive TNF-*α* expression. By comparison, the groups administered PCFEAMS demonstrate significantly fewer TNF-*α* markers.

**Figure 9:**
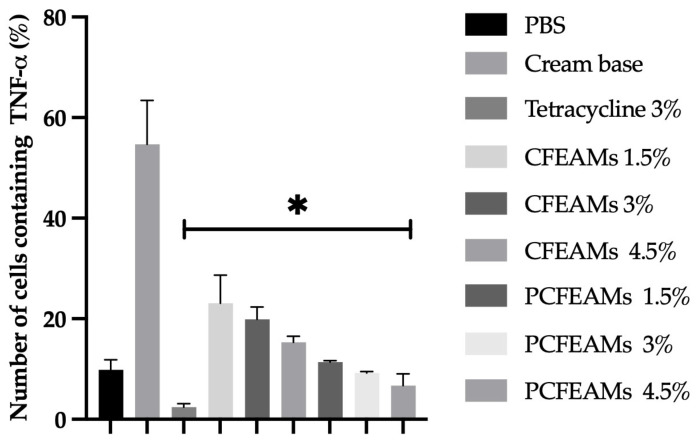
Immunohistochemical of TNF-*α* expression in skin macrophage cells. The bars represent cells positive for TNF-*α* (indicated by brown DAB chromogen staining). Data are presented as mean ± SD from triplicate experiments (*n* = 3). Statistical significance was determined by One-Way ANOVA followed by Dunnett’s multiple comparison test. The asterisk (*) denotes a significance difference (*p* < 0.05) compared to the negative group (basis).

According to the data in Figure 9, the experimental group receiving tetracycline cream showed a substantially lower percentage of TNF-*α* compared to other groups. Furthermore, the animals treated with the cream base exhibited the highest percentage of TNF-*α* among all test subjects. When comparing CFEAMS and PCFEAMS, the reduction in TNF-*α* percentage was notably greater in the groups receiving the phytosome cream. Ultimately, statistical analysis yielded a significant value of 0.0001 (*p* < 0.05).

**Figure 10:**
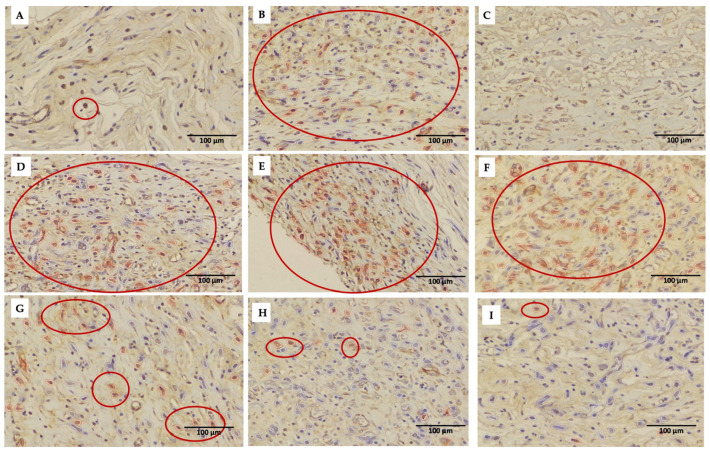
Immunohistochemical images of IL-6 expression in experimental groups treated with (**A**) PBS, (**B**) cream base (negative control), (**C**) 3% tetracycline cream (positive control), different concentrations of CFEAMS at (**D**) 1.5%, (**E**) 3%, and (**F**) 4.5%, and PCFEAMS at concentrations of (**G**) 1.5%, (**H**) 3%, and (**I**) 4.5%, at 400× magnification. The presence of these brown stains indicates the specific sites (marked with a red circle) where the IL-6 antibody has successfully bound.

Immunohistochemical analysis (see Figure 10) of the experimental group treated with the cream base (acting as the negative control) revealed the presence of brown staining (marked with a red circle) across various cells. This specific coloration indicates the precise locations where the IL-6 antibody successfully bound to the tissue. When evaluated comprehensively, this group exhibited the most extensive IL-6 expression among all subjects. Conversely, the groups administered PCFEAMS displayed a significantly reduced presence of IL-6 markers.

**Figure 11:**
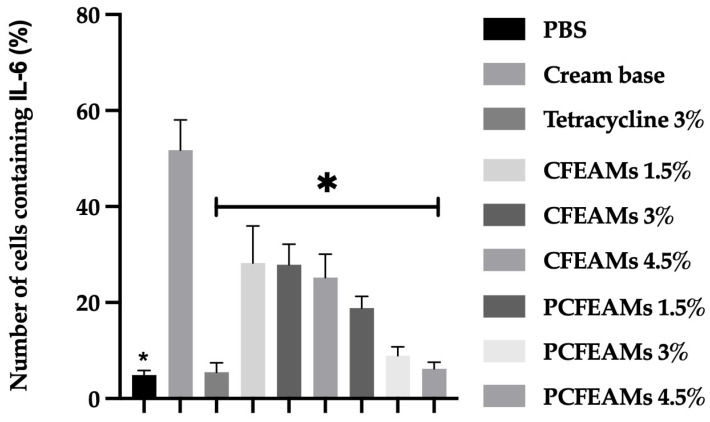
Immunohistological quantification of IL-6 expression in skin macrophage cells. The bars represent cells positive for IL-6 (indicated by brown DAB chromogen staining) Data are expressed as mean ± SD (*n* = 3). Statistical significance was analyzed using One-Way ANOVA followed by Dunnett’s multiple comparisons test. Asterisks indicate significant differences compared to the basis group (* *p* < 0.05).

The percentage of IL-6 depicted in Figure 11 for the experimental group treated with 3% tetracycline cream shows a substantially lower IL-6 level compared to the other groups. Conversely, the animals administered the cream base exhibited the highest percentage of IL-6 among all test subjects. When comparing the groups treated with CFEAMS and PCFEAMS, the reduction in IL-6 percentage was more pronounced in those receiving the phytosome cream. Accordingly, statistical analysis yielded a significant value of 0.0001 (*p* < 0.05).

The immunohistochemistry results illustrated in Figure 10 for the experimental group treated with the cream base (serving as the negative control) reveal the presence of brown staining across various cells. This specific coloration indicates the precise locations where the IL-6 antibody has successfully bound to the tissue. When evaluated comprehensively, this group exhibited the most extensive IL-6 expression among all subjects. Conversely, the groups administered PCFEAMS displayed a significantly reduced presence of IL-6 markers.

### 2.9. Statistical Analysis

The data were presented and analyzed using a one-way analysis of variance (ANOVA) via GraphPad Prism 11.0.1. For all tests, a *p*-value of less than 0.05 was considered statistically significant. Ultimately, all data are presented as the mean ± SD, derived from three independent replicates for each experiment.

## 3. Discussion

This research evaluated the antibacterial activity of the ethanol extract and various fractions of *M. speciosa* Blume fruit against *C. acnes* while simultaneously developing phytosome formulations to enhance therapeutic effectiveness. Notably, the ethyl acetate fraction exhibited the highest activity among all tested fractions, producing the largest inhibition zone and reaching a relative activity of 93.5% compared to tetracycline (see Table 1). Based on these potent results, phytosomes were synthesized specifically from the ethyl acetate fraction. Characterization data revealed that the ethyl acetate fraction phytosomes were significantly smaller (256.70 ± 1.10 nm) than those derived from the ethanol extract (835.96 ± 122.63 nm). Furthermore, the negative zeta potential of the ethyl acetate phytosomes (−82.7 ± 1.37 mV) indicates excellent electrostatic stability (see Table 2). Regarding long-term integrity, entrapment efficiency remained stable and was not significantly influenced by storage temperature or duration. Morphological observation via TEM confirmed that these phytosomes possess a spherical surface (see Figure 1). Additionally, FTIR analysis indicated that no new covalent bonds were formed. Instead, weak molecular interactions, such as hydrogen bonding, occurred between the phospholipids and the ethyl acetate fraction (see Figure 2). In terms of delivery, PCFEAMS demonstrated superior penetration of active compounds compared to CFEAMS, with absorption increasing alongside time and concentration (see Figure 3). Results from the in vivo antibacterial assays against *C. acnes* showed that PCFEAMS significantly reduced the number of bacterial colonies (see Figure 4). Likewise, histological analysis indicated a more effective reduction in inflammatory cells in the PCFEAMS groups than in the cream base group (see Figure 5 and Figure 6), which was further supported by a marked decrease in TNF-*α* and IL-6 levels (see Figure 7, Figure 8, Figure 9 and Figure 10).

Antibacterial testing was performed on the ethanol extract and three fractions of varying polarity, namely *n*-hexane (non-polar), ethyl acetate (semi-polar), and water (polar). According to established inhibition zone classifications, a zone of less than 9 mm is considered inactive, 9 to 12 mm is partially active, 13 to 18 mm is active, and greater than 18 mm is classified as highly active [21]. The results indicate that the extract and all fractions produced a strong antibacterial effect with inhibition zones exceeding 10 mm. Consequently, the extract and the ethyl acetate fraction at a concentration of 50 mg/mL provided active/strong inhibition. However, their minimum effective concentrations varied. Specifically, the *n*-hexane fraction showed antibacterial effects starting at 25 mg/mL, while the ethanol extract and ethyl acetate fraction began inhibiting *C. acnes* at just 6.25 mg/mL. By comparison, the water fraction required a concentration of at least 12.5 mg/mL to be effective. Although these results are impressive, the resistance generated was lower than that of the tetracycline positive control. Interestingly, when compared to previous studies on the ethanol extract of *M. speciosa* Blume leaves, which showed weak inhibition (4.94 ± 1.38 mm at 50.00 mg/mL), these findings suggest that the fruit of the plant inhibits *C. acnes* far more effectively [22]. Accordingly, phytosome formulations were developed using the extract and fraction that demonstrated active effects at the lowest concentrations, namely the ethanol extract and the ethyl acetate fraction.

Based on the antibacterial activity results demonstrating the potent effects of the ethanol extract and the ethyl acetate fraction, a phytosome-based delivery system was subsequently formulated to enhance the penetration of active compounds. Characterization of these phytosomes involved evaluating the physicochemical properties and the overall stability of this nano-system. Characterization results showed that the EAFMS-phytosome was significantly more stable than the extract-phytosome. The high zeta potential of the EAFMS-phytosome provides a sufficient repulsive force to prevent vesicle coalescence, whereas the low zeta potential of the extract-phytosome suggests an unstable system prone to rapid degradation. Consequently, the ethyl acetate fraction was identified as the most suitable candidate for phytosome complexation, and further studies were focused exclusively on this formulation.

According to the characterization results for the extract and the fraction, the ethyl acetate fraction yielded significantly smaller particle sizes. Notably, vesicles with a diameter of 600 nm or greater cannot deliver encapsulated materials into the deeper layers of the skin. Instead, such vesicles tend to remain within the stratum corneum and may form a lipid film on the skin surface upon drying [23]. Evaluation of phytosome stability during storage is generally performed by monitoring physicochemical parameters, specifically particle size, polydispersity index, and zeta potential. Studies indicate that significant fluctuations in these parameters during storage can signal instability within the phytosome system [18,24].

The ethyl acetate fraction resulted in significantly smaller phytosome particle sizes. Stability testing during 90 days of storage at 25 °C and 40 °C showed a minimal increase in particle size, confirming that this nanoparticle system is stable and resistant to significant macroscopic aggregation. This characteristic is supported by the polydispersity index (PDI) value which remained within the prerequisite limits for a lipid delivery system (reaching 0.53 at day 90), reflecting a homogeneous particle size distribution [25]. PDI is an indicator of particle size uniformity (the closer it is to 0, the more uniform). PDI values below 0.5 are generally considered to have a relatively good size distribution and are acceptable for lipid/nanoparticle delivery systems [26].

Generally, a zeta potential value above +30 mV or below −30 mV signifies excellent physical stability for colloidal systems, including nanoparticles, due to strong inter-particle repulsion that prevents aggregation. Conversely, if particles possess a low zeta potential value, they tend to clump or aggregate, resulting in an unstable delivery system. Furthermore, if the system maintains a large positive or negative zeta potential, the particles will likely repel one another, demonstrating a lack of affinity for clumping [27,28]. The negative zeta potential values obtained in this study are attributed to the negative phosphate groups within the phospholipids, which orient toward the outer layer of the phytosome complex [29,30].

Entrapment efficiency becomes a critical parameter for evaluating nano-drug delivery systems, as it measures the percentage of active compounds successfully encapsulated within the carrier system relative to the total amount utilized [31]. Essentially, this metric reflects the ability of the phospholipid complex to bind active phytochemical compounds from plant extracts. A high entrapment efficiency value exceeding 80% signifies an efficient formulation with minimal drug wastage during the preparation process [32,33]. Based on the recorded data, the phytosome formulation achieved a remarkably high entrapment efficiency of 89%. Furthermore, this stability at 25 °C indicates the formation of an effective phytosome system that remains relatively insensitive to temperature fluctuations [34]. Soy lecithin, utilized as the polymer in these phytosomes, is a natural phospholipid derived from soybeans with a unique amphiphilic structure consisting of a hydrophilic phosphate group and lipophilic fatty acid chains [35]. Moreover, soy lecithin forms complexes with bioactive compounds via hydrogen bonding with phenolic hydroxyl groups. This observation aligns with established research regarding the structure–activity relationship between phospholipids and polyphenols, which suggests that the amphiphilic nature of phospholipids enhances polyphenol solubility through encapsulation while protecting them from environmental oxidation [36].

Once the physical stability and particle size of the phytosome formulation were confirmed to meet target specifications, structural analysis via FTIR was conducted to investigate the molecular interactions between the active compounds and the vesicle components. Phospholipids, as the primary constituent of lecithin, possess characteristic functional groups such as esters, amines, and phosphates [37]. Each of these functional groups displays specific absorption bands within the FTIR spectrum. Consequently, the FTIR analysis of soy lecithin, as shown in Figure 3, reveals several absorption peaks across various regions. The spectrum exhibits a characteristic peak at 3386.73 cm^−1^ resulting from O-H stretching vibrations and hydroxyl groups. Additionally, peaks at 2925.25 and 2854.28 cm^−1^ are caused by the −CH stretching of methylene groups within long fatty acid chains. Other notable peaks appear at 1648.62 and 1233.34 cm^−1^, attributed to C-O stretching in fatty acid esters. Finally, the stretching detected in the range of 1067.40 to 1165.85 cm^−1^ confirms the presence of the phosphate groups.

FTIR analysis of the ethyl acetate fraction reveals O-H group stretching detected at 3388.12 cm^−1^ with strong intensity. Additionally, the aryl ketone C=O group stretching appears at 1609.62 cm^−1^, also exhibiting strong intensity. Aromatic ring C=C stretching bands are identified at 1613.09, 1514.45, and 1445.76 cm^−1^. The band at 1243.20 cm^−1^ is attributed to C-O stretching within the aryl ether ring, while aromatic C-H stretching emerges at 770.99 cm^−1^ with weak intensity. FTIR spectroscopy of the soy lecithin and ethyl acetate fraction complex indicates several absorption peaks across various regions (see Figure 3). This analysis was specifically performed to identify molecular interactions between the soy lecithin and the EAFMS. Notably, no new peaks emerged within the physical phytosome mixture.

Small shifts in the stretching vibration peaks related to −P=O at 1184.77 cm^−1^ in the phosphate group of the phospholipid molecule were observed in the day 0 phytosome spectrum. Furthermore, shifts occurred in the −C=O carbonyl ketone group at 1742.90 cm^−1^. The sharp peak associated with free O-H stretching vibrations (3386.43 cm^−1^) also shifted during the initial preparation, which points to the formation of hydrogen bonds between the free O-H groups in the EAFMS and the phospholipids. After 90 days of storage, the phytosome spectrum displayed a −P=O stretching vibration peak at 1194.91 cm^−1^, while the free O-H stretching vibration shifted to a wavenumber of 3424.59 cm^−1^. Based on these observations, it is evident that no new conjugative bonds were formed between the ethyl acetate fraction and the phospholipids. Instead, the data suggest the presence of weak intermolecular interactions, such as hydrogen bonding between the hydroxyl groups of the ethyl acetate fraction and the carbonyl and phosphoryl groups of the phospholipids. These results align closely with previous reports on silybin phytosome complexes, which also showed no new chemical bonds but rather weak interactions like hydrogen bonding [17,38].

The DSC graph provides a solid structural picture of the formation of a phytosome complex. This formation is characterized by the loss of the original crystalline nature of the ethyl acetate fraction and the emergence of a new, characteristic amorphous thermal profile due to molecular bonding with soy lecithin [39].

Through Franz diffusion cell testing, the phytosome formulation (PCEAFMS) demonstrated a significantly higher cumulative penetration percentage than the free fraction form (CFEAMS). The nanoparticle size and low interfacial tension effectively facilitate the penetration of active ingredients into deeper skin layers, while ensuring a controlled and sustained release profile [40]. Furthermore, nano-sized phytosomes enhance absorption and bioavailability, improve the solubility of hydrophobic active ingredients, and ensure rapid delivery for many drugs [41,42]. These observations align with findings by Hendawy et al., which showed that quercetin release from a phytosome complex was significantly higher than in non-phytosomal formulations [19].

These release characteristics correlate directly with in vivo test performance in an animal model of *C. acnes*-induced acne. The phytosome formulation significantly reduced the *C. acnes* bacterial population compared to the control group and the standard fraction due to its ability to accumulate in lipid-rich sebaceous follicles.

The therapeutic effects of phytosomes extend beyond microbial inhibition by mitigating secondary tissue damage caused by the inflammatory cascade. Histopathologically, this phytosome system normalizes the structure of sebaceous follicles and drastically reduces the infiltration of inflammatory cells such as polymorphonuclear filaments (PMNs), monocytes, and lymphocytes. Tissue healing is accelerated by stimulating fibroblast proliferation for new collagen synthesis and increasing blood vessel density for essential nutrient supply [43,44,45].

Bacterial quantification results indicate that the phytosome fraction cream reduces the bacterial count more effectively than the fraction cream alone. Evidence suggests that the development of phytosomes provides superior efficacy because small particle sizes enhance the absorption of active ingredients into the skin. Consequently, these formulations achieve a greater reduction in bacterial load compared to the control group [46]. These findings align with research regarding the antimicrobial utilization of *Annona squamosa* and *Cinnamomum tamala* extracts, where phytosome creams produced a more significant decrease in bacteria than Povidone-iodine ointment [47].

In vivo testing results further demonstrate that the phytosome formulation provides a substantial reduction in the *C. acnes* population compared to the control and CFEAMS groups. To understand the profound therapeutic impact, one can observe the histopathological images, where a decrease in polymorphonuclear (PMN) cell infiltration and the normalization of sebaceous follicle structures are evident. Accordingly, the effect of phytosomes is not limited to microbial inhibition but also encompasses the mitigation of secondary tissue damage caused by inflammation.

Descriptively, PCFEAMS formulations appeared to show a trend toward lower inflammation indicators compared to CFEAMS; however, these histological observations were based on semi-quantitative scoring and were not confirmed by formal inferential statistical analysis. Therefore, these between-group differences should be interpreted with caution [48,49].

Pathological parameters indicate that the topical application of PCFEAMS and tetracycline cream reduces inflammatory tissue while simultaneously increasing epithelial cells and fibroblasts compared to control rats treated with the cream base or CFEAMS. Histological analysis of the PBS group reveals almost no inflammatory cell infiltration and a relatively organized collagen matrix. This specific observation represents healthy skin without acne lesions, meaning no active healing process is occurring [50]. In contrast, the group treated with the cream base shows dense neutrophil infiltration in the dermis and disorganized collagen fibers. Biologically, in cases of inflamed nodulocystic acne or papulopustules, the rupture of follicle walls containing sebum, keratin, and bacteria triggers the release of inflammatory mediators like TNF-*α* and IL-6. Consequently, neutrophils and macrophages migrate massively to the lesion. Because tissue damage is serious during this phase, the risk of atrophic scarring is also significant [51,52].

Regarding the tetracycline cream group, inflammatory cell infiltration remains present but appears more dispersed. Tetracycline was employed as a positive control because it functions not only as an antibacterial agent against *C. acnes* but also exhibits specific anti-inflammatory properties by inhibiting matrix metalloproteinases (MMPs) and reducing the production of inflammatory mediators. Consequently, in the tetracycline-treated group, both the bacterial count and the intensity of inflammation were reduced through these dual mechanisms. Accordingly, the bacterial count and the intensity of inflammation are reduced [53,54,55]. The CFEAMS groups appeared to exhibit higher residual inflammatory cell counts relative to PCFEAMS, suggesting that the phytosome delivery system may offer additional anti-inflammatory benefit; however, in the absence of statistical testing of histological data, this observation remains descriptive and requires confirmation in adequately powered studies.

This suggests that the phytosome delivery system at various concentrations can shorten the inflammatory phase and lead to a more controlled remodeling phase. During this transition, collagen matures from type III to type I, while scar tissue becomes thinner and more organized [34,39]. Clinically, this translates to lesions subsiding faster, reduced post-acne erythema, and a lower risk of raised or pitted scars [56]. The core mechanism of acne wound healing, as correlated with the histological findings, involves three distinct stages. First, the inflammatory phase occurs when follicular rupture and *C. acnes* induce dense neutrophils, as seen in the base and low-concentration fraction groups. Second, the proliferation or granulation phase begins once bacterial counts and inflammatory mediators decrease, and new blood vessels dominate. This is evident in the tetracycline, 4.5% fraction, and phytosome groups. Finally, the remodeling phase represents the most effective therapeutic outcome, particularly in the phytosome groups. Histology confirms that phytosomes accelerate the transition to tissue with more organized collagen and fewer inflammatory cells. Correspondingly, this leads to superior healing and a reduced risk of scarring [57,58,59]. These findings align with research on topical eugenol phytosomes and mupirocin ointment, which also showed significant reductions in inflammation and increases in epithelial tissue and fibroblasts [36].

*C. acnes* is a bacterium predominantly colonizing hair follicles and sebaceous glands. Under normal circumstances, *C. acnes* assists in maintaining a healthy skin microbiome. Nevertheless, this equilibrium can be disrupted under specific conditions. Factors such as increased sebum production and follicular hyperkeratosis often lead to bacterial overgrowth. This proliferation subsequently triggers an inflammatory response in the skin, which contributes significantly to the formation of acne lesions [60,61,62]. As *C. acnes* multiplies, it releases various substances recognized by the skin’s immune cells, marking the starting point of the inflammatory cascade.

Specifically, *C. acnes* possesses pathogen-associated molecular patterns (PAMPs), such as cell wall components and bacterial DNA, which are recognized by toll-like receptors (TLRs). Studies have shown that *C. acnes* primarily activates TLR2 and TLR4 on monocytes, macrophages, and keratinocytes. TLR activation is a crucial step in initiating inflammatory signaling pathways. Once PAMPs from *C. acnes* interact with these receptors, intracellular signaling pathways are activated and trigger the production of pro-inflammatory cytokines. TLR activation by *C. acnes* activates nuclear factor kappa beta (NF-*κβ*). NF-*κβ* then translocates to the cell nucleus and induces the expression of genes responsible for the production of these inflammatory cytokines [60,63].

Among the pro-inflammatory cytokines produced are tumor necrosis factor alpha (TNF-*α*) and interleukin 6 (IL-6). While TNF-*α* plays a central role in triggering and sustaining the inflammatory response, IL-6 is responsible for regulating immune reactions [64,65]. In acne conditions, high levels of TNF-*α* maintain the recruitment of neutrophils and monocytes while increasing the expression of matrix metalloproteinases (MMPs) and matrix degradation. Accordingly, this elevates the risk of scarring and post-inflammatory hyperpigmentation [66,67].

Immunohistochemical examination results showed that PCFEAMS derived from *M. speciosa* fruit produced lower levels of TNF-*α* and IL-6. This occurs because the structure of phytosomes is very similar to cell membranes, thereby increasing the ability of plant molecules to penetrate the skin layer. This phospholipid structure also facilitates the active compounds’ absorption in greater quantities by TNF-*α* and IL-6-producing skin cells [68]. Furthermore, this phospholipid arrangement allows active compounds to be absorbed more easily and in greater quantities by the skin cells responsible for producing TNF-*α* and IL-6 [69,70,71].

Phytosomes improve interactions with skin cell membranes, enabling active compounds to enter cellular mechanisms directly and suppress cytokine production more effectively [72]. With a larger amount of active compounds reaching the target, the suppression mechanism operates more efficiently, resulting in lower production of TNF-*α* and IL-6 [73,74].

In the context of acne wound healing, reducing the expression of these cytokines accelerates the skin’s transition to the proliferative and remodeling phases. Consequently, clinical healing of acne lesions is accelerated, post-acne erythema is reduced, and scar tissue quality improves [66,67,75]. These findings align with existing research showing that phytosomes from *Zea mays* L. and ginger extracts exhibit superior activity in lowering TNF-*α* [48,76,77]. Similarly, phytosomes from tetrahydrocurcumin gel enhance penetration into deeper skin layers, thereby reducing TNF-*α* and IL-6 levels significantly [78,79].

Several limitations of this study should be acknowledged. First, the in vivo experiments were conducted with a relatively small number of animals per group (*n* = 3). Second, the study design did not incorporate observer bias in the semi-quantitative scoring of inflammatory cell infiltration and tissue remodeling parameters. Third, although three concentration of PCFEAMS (1.5%, 3.0%, and 4.5%) were tested for antibacterial activity, a formal dose–response relationship was not establish for the anti-inflammatory markers TNF-*α* and IL-6; thus the optimal therapeutic concentration remains to be determined through systemic dose-escalation studies. The in vivo acne model employed in this study, although widely used, may not fully recapitulate the multifactorial pathophysiology of human acne vulgaris, which involves hormonal, microbiome, and genetic components beyond the scope of the current experimental design.

To address the limitations identified above and to advance the translational potential of the PCFEAMS delivery system, several directions for future research are recommended. First, subsequent in vivo studies should employ a larger sample size. Second, future experimental designs should incorporate blinded assessment protocols. Third, a systemic dose-escalation study encompassing a broader concentration range of PCFEAMS is needed.

## 4. Materials and Methods

### 4.1. Materials and Equipment

The materials utilized throughout this research consisted of 96% ethanol (Brataco, Bandung, Indonesia), methanol p.a. (Merck, Singapore), AQUA PRO Injection (Kimia Farma, Jakarta, Indonesia), and distilled water or Aquadest (Kimia Farma, Jakarta, Indonesia). Additionally, the formulation involved PEG 4000 24% (Merck), 12% propylene glycol (Brataco), 25% stearic acid (Brataco), 3.2% cetyl alcohol (Merck), 1.5% triethanolamine (Merck), 0.32% methylparaben (Merck), and 0.3% potassium hydroxide (Sigma-Aldrich, Darmstadt, Germany).

Regarding the laboratory apparatus, the study employed a rotary evaporator (Heidolph, Schwabach, Germany) and a hot plate magnetic stirrer (Thermo Fisher Scientific, Singapore). For analytical purposes, the process utilized a particle size analyzer (Horiba Scientific SZ-100, Kyoto, Japan), a double-beam UV 1800 spectrophotometer (Shimadzu, Sanjo, Japan), and 10–100 µL micropipettes (Acura 825, Tokyo, Japan). Furthermore, measurements and processing were conducted using a pH meter and micropump (Hanna Instruments, Shenzhen, China), a centrifuge (Labnet, Shenzhen, China), and a transmission electron microscope (Jeol JEM-1400, Tokyo, Japan). To evaluate the release profiles, the setup incorporated a Franz diffusion cell (Masterflex, Houston, TX, USA) along with various plastic labware (Thermo Fisher Scientific, Waltham, MA, USA).

### 4.2. Extraction and Fractionation

Dried powder from *M. speciosa* Blume fruit (900 g) was extracted through the maceration method using 96% ethanol at a solvent-to-material ratio of 1:10 (*w*/*v*). Specifically, the powder was fully submerged in ethanol within a macerator vessel and kept at room temperature (25 ± 2 °C) for 24 h. This maceration process was repeated three times with fresh solvent for each cycle to maximize the extraction of bioactive constituents. After each cycle, the extract was filtered using Whatman No. 1 filter paper. The combined filtrates were then concentrated under reduced pressure using a rotary evaporator at 50 °C, resulting in a thick ethanolic extract.

Subsequently, a portion of the ethanolic extract was dissolved in 400 mL of distilled water for liquid–liquid partitioning. The aqueous solution was transferred to a separatory funnel and extracted with *n*-hexane (3 × 400 mL) to obtain the *n*-hexane fraction. The *n*-hexane layers were collected and evaporated at 40 °C using a rotary evaporator to produce the final *n*-hexane fraction 8.5% *w*/*w*). Afterward, the remaining aqueous layer was further extracted with ethyl acetate (3 × 400 mL) to yield the ethyl acetate fraction (8% *w*/*w*), which was also evaporated under reduced pressure. The final aqueous phase was collected as the water fraction 56% *w*/*w*. Each fraction was concentrated until dry and stored in tightly sealed dark containers [80,81].

### 4.3. Determination of Total Flavonoid Content

The total flavonoid content within extracts and fractions was determined using the aluminum chloride colorimetric method [82]. Initially, extracts and fractions were prepared at a concentration of 1 mg/mL and adjusted to a 1 mL volume with methanol. This solution was mixed with 4 mL of distilled water, followed by the addition of 0.3 mL of 5% NaNO_2_ solution. After a five-minute incubation period, 0.3 mL of 10% AlCl_3_ was introduced, and the mixture remained undisturbed for an additional six minutes. Subsequently, 2 mL of 1 mol/L NaOH solution was added, and the final volume of the mixture was adjusted to 10 mL. Following a final 15 min standing period, the absorbance was measured at 510 nm. Accordingly, the total flavonoid content was calculated using a calibration curve, with results expressed as milligrams of quercetin equivalents (QEs) per gram of dry weight [83].

Determination of total flavonoid content using quercetin as a comparison standard, obtained in ethanol extract 6.16 ± 0.24 mg QE/g, *n*-hexane fraction 29.23 ± 0.10 mg QE/g, ethyl acetate fraction 40.25 ± 0.958 mg QE/g, and water fraction 3.97 ± 0.04 mg QE/g (R^2^ = 0.996).

### 4.4. Antibacterial Activity Assay of Extracts and Fractions

The antibacterial efficacy of the extracts and ethyl acetate fractions from *M. speciosa* Blume fruit against *C. acnes* ATCC 6919 was evaluated via the agar diffusion method using the perforation technique. To begin the procedure, extracts and fractions were prepared at concentrations of 6.25, 12.5, 25, and 50 mg/mL, dissolved in 1.0% *v*/*v* dimethyl sulfoxide (DMSO). Petri dishes were filled with 20 mL of Mueller–Hinton Agar (MHA) medium and allowed to solidify. Next, the *C. acnes* suspension was spread evenly across the surface using a sterile swab. The plates were kept at room temperature for 30 min to allow the inoculum to dry. Subsequently, holes were created using a sterile 5.6 mm diameter perforator, and 50 μL of each extract and fraction was added to the respective wells. The plates were then left at room temperature for 1 h to facilitate the pre-diffusion of the active compounds before being incubated at 37 °C for 18–24 h under anaerobic conditions. Systematically, 50 μL of each extract and fraction was added to the respective wells. For comparison, a 1.0% DMSO solution served as the negative control, while tetracycline at 30 μg/mL was utilized as the positive control. The plates were then incubated at 37 °C for 18–24 h. Ultimately, the antibacterial activity was determined by measuring the diameter of the clear inhibition zones surrounding each well [84].

### 4.5. Preparation and Stability Evaluation of EAFMS-Phytosome Complexes

The EAFMS-phytosome complexes were synthesized via the antisolvent precipitation method. To initiate this process, a mixture of the extract/EAFMS and soy lecithin was dissolved in 20 mL of dichloromethane and refluxed for 3 h at 50 °C. Subsequently, the solution was concentrated to a 5 mL volume. Following this concentration, 20 mL of *n*-hexane was introduced dropwise at a rate of 2 mL/min while the mixture was stirred continuously at 1000 rpm. The ratio of soy lecithin to the ethyl acetate fraction was maintained at 1:3 (1000 mg to 3000 mg), and stability testing was performed over 30, 60, and 90 days.

### 4.6. Phytosome Characterization Procedure

#### 4.6.1. Entrapment Efficiency

Entrapment efficiency (*EE*) was evaluated by dissolving the phytosomes in methanol and subjecting them to centrifugation at 13,000 rpm for 20 min at 4 °C. Precisely 1 mL of the supernatant was collected, and its absorbance was measured using a double-beam UV 1800 spectrophotometer (Shimadzu, Sanjo, Japan). Ultimately, the entrapment efficiency was calculated according to the following formula.

$$EE\left(\%\right)=\frac{Total\;flavonoid\;content\;in\;phytosome-Total\;flavonoid\;content\;in\;supernatant}{Total\;flavonoid\;content\;in\;phytosome}\times 100(\%)$$

#### 4.6.2. Particle Size, Polydispersity Index, and Zeta Potential

Phytosome characterization further included the assessment of particle size and distribution using a particle size analyzer (PSA) via the dynamic light scattering (DLS) method. These measurements were conducted using a Horiba Scientific SZ-100 (Kyoto, Japan). For the analysis, samples were diluted tenfold and measured at room temperature [85,86].

#### 4.6.3. Morphological Observation of the Phytosomes via Transmission Electron Microscopy (SEM)

The morphological characteristics of phytosomes were evaluated using a transmission electron microscope (TEM) (Jeol JEM-1400, Tokyo, Japan). One drop of the phytosome dispersion was placed onto a carbon-coated copper grid and stained with a 2% (*w*/*v*) uranyl acetate solution. Excess stain was removed using filter paper. After air-drying the grid, the specimens were viewed under TEM at an accelerating voltage of 100 kilovolts (kV) [48].

#### 4.6.4. In Vitro Skin Permeation Study

An in vitro skin permeation study was performed in triplicate for each formulation using a modified circulating Franz diffusion system. The system consists of a diffusion cell, a peristaltic pump (Masterflex), and a continuously stirred reservoir (glass) media. The dorsal skin of male Wistar rats was used. After hair removal, the skin was excised and the subcutaneous fat was carefully removed to obtain full-thickness skin. Skin integrity was verified visually for any defects and pre-equilibrated in the receptor media for 1 h before the application of the cream. Only intact skin samples were used to ensure consistent permeation barriers. To begin the procedure, 1 g cream was applied onto prepared rat skin with a surface diameter of 3 cm. The diffusion cell was integrated into a closed-loop circulation system connected to a larger external reservoir (beaker glass) to achieve a total receptor phase volume of 300 mL of phosphate buffer at pH 7.4, maintained at a steady temperature of 37 °C. Subsequently, 3 mL samples were withdrawn at intervals of 5, 10, 15, 30, 60, 90, 120, 180, 240, 270, 300, 360, 420, and 480 min. Precisely, 3 mL of fresh phosphate buffer (pH 7.4) was replaced after each withdrawal to maintain sink conditions. Absorbance was measured using a UV-visible spectrophotometer at a wavelength of 255 nm, determined through quercetin wavelength calibration. These measurements were conducted against a phosphate buffer blank. This testing evaluated the cream of ethyl acetate fractions of *M. speciosa* (CFEAMS) and phytosome cream of ethyl acetate fractions of *M. speciosa* (PCFEAMS) at concentrations of 1.5%, 3%, and 4.5% [87,88].

The cumulative penetration data was expressed as a percentage relative to the initial applied dose of the active fraction. The total applied dose (*D*_0_) was determined quantitatively based on the weight of the cream (1 g) and respective concentration, yielding 15 mg, 30 mg, and 45 mg of the active fraction for the 1.5%, 3% and 4.5% formulations, respectively. Consistent with Fick’s First Law of Diffusion under finite dose conditions, the cumulative permeation percentage (%*P*) was calculated by normalizing the cumulative amount of drug per unit area (*Q_t_*/A) against the initial applied dose per unit area (*D*_0_/A), as shown in the following equation:

$$\%P=\;\frac{Q_{t}}{D_{0}}\;\times \;100\%$$

where *Q_t_* is the cumulative mass of the active compound in the receptor medium (μg) at time (*t*), and *D*_0_ is the initial applied dose (μg). Because the effective diffusional surface area (=7.07 cm^2^, derived from the 3 cm diameter) is constant for both the permeated amount and the applied dose, it mathematically cancels out [39].

#### 4.6.5. Stability Testing

Stability assessments were performed under both long-term (25 °C ± 2 °C/60% ± 5% RH) and accelerated (40 °C ± 2 °C/75% ± 5% RH) conditions. Samples were systematically collected at intervals of 0, 30, 60, and 90 days. To assess the stability profile, particle size, polydispersity index, and zeta potential were measured. Additionally, the entrapment efficiency was evaluated following each storage interval to detect any significant changes in formulation integrity [89,90,91].

#### 4.6.6. FTIR Characterization

FTIR analysis (Shimadzu, Sanjo, Japan) was performed to investigate the chemical interactions between the ethyl acetate fraction and soy lecithin. Specifically, the ethyl acetate fraction, soy lecithin, and the ethyl acetate fraction phytosomes at day 0 and day 90 were individually blended with potassium bromide (KBr). Each sample was thoroughly dried using a sodium lamp and ground into a fine powder with the KBr. The dry mixture was then compressed into a thin pellet using a hydraulic press. Scanning was executed across a range of 400 to 4000 cm^−1^ with a resolution of 2 cm^−1^ [92].

#### 4.6.7. Differential Scanning Calorimetry Procedure

The thermal behavior of ethyl acetate fraction, soy lecithin, and physical mixture was analyzed to verify the formation of the phytosomal complex. The thermal analysis was carried out using differential scanning colorimetry (DSC-60 Plus; Shimadzu, Japan). The samples were heated from 20 to 400 °C at a 10 °C/min heating rate.

### 4.7. In Vivo Antibacterial Activity Testing of Phytosomes Against C. acnes

#### 4.7.1. Acclimatization of Experimental Animals

The animal trials were supervised and approved by the Ethics Committee of the Faculty of Pharmacy at Jenderal Achmad Yani University, registration code number 10001/KEP-UNJANI/III/2025, approved on 28/March/2025. Experimental subjects consisted of male Wistar strain white rats weighing between 150 and 200 g, aged 15 weeks, obtained from PT. Biofarma, Indonesia. These rats were adapted for seven days within a controlled environment featuring a 12 h light/dark cycle. Temperature was maintained at 25 ± 3 °C, while humidity was kept between 50% and 60%. Throughout this acclimatization period, the rats had unrestricted access to food and clean water. Subsequently, *C. acnes* was induced intradermally into the subjects.

#### 4.7.2. Experimental Animal Grouping

The rats were randomly divided into nine distinct groups, each comprising three individuals. The sample size (*n* = 3 per group) was determined based on Federer’s formula and previous established protocols for in vivo acne models to ensure statistical validity while adhering to the principle of the 3Rs (Replacement, Reduction, and Refinement). Randomization was performed using a computer-generated random sequence to allocate animals to treatment groups, ensuring an unbiased distribution. All animals were monitored daily during the 7-day acclimatization period, and only those showing no signs of distress or illness were included; no animals were excluded from the study after the commencement of the experiments. Groups 2 to 9 underwent *C. acnes* induction followed by therapy as detailed in the subsequent explanation. Group 1 normal rats received only a sterile PBS injection (sterility control). Group 2, serving as the negative control, received the cream base. Group 3 was treated with a 3% tetracycline cream. Groups 4, 5, and 6 were administered CFEAMS at concentrations of 1.5%, 3%, and 4.5%, respectively. Correspondingly, Groups 7, 8, and 9 received PCFEAMS at concentrations of 1.5%, 3%, and 4.5%.

#### 4.7.3. Test Bacteria

*C. acnes* ATCC 6919 was sourced from PT. Agritama Sinergi Inovasi, West Java, Indonesia. The bacteria were cultured on reinforced clostridium medium (RCM) agar under anaerobic conditions at 37 °C for three days. Following this, colonies were suspended in PBS and adjusted to an absorbance of 1.5 at a wavelength of 600 nm. For *C. acnes*, an *OD*_600_ of 1 corresponds to 5 × 10^8^ CFU·mL^−1^ This bacterial suspension was utilized for the experiment. Each rat received an intradermal injection of 0.25 mL of the suspension.

#### 4.7.4. In Vivo Antibacterial Activity Evaluation

The in vivo antibacterial efficacy of the ethyl acetate fraction and the ethyl acetate fraction phytosomes from *M. speciosa* Blume fruit against *C. acnes* was assessed using a rat model via intradermal back injections. To induce infection, 0.25 mL of *C. acnes* (7.5 × 10^8^ CFU/mL^−1^) was injected intradermally into the dorsal region of the rats for three consecutive days. Subsequently, tetracycline cream, the ethyl acetate fraction, and the phytosome formulations (composed of 24% *w*/*w* PEG 4000, 12% propylene glycol, 25% stearic acid, 3.2% cetyl alcohol, 1.5% triethanolamine, 0.32% methylparaben, and 0.3% potassium hydroxide) were applied to the lesion sites twice daily. After five days, the rats were euthanized to facilitate bacterial counting and skin biopsies. Bacterial quantification involved rinsing the skin area and homogenizing the tissue in 1 mL of sterile PBS. The resulting homogenate was diluted from 1:10 to 1:10^6^ in PBS, after which 10 μL of each dilution was incubated at 37 °C for three days to measure the *C. acnes* colony-forming units (CFU) [93,94].

#### 4.7.5. Histology and Immunohistochemistry

Skin tissue samples collected from the experimental animals were fixed in 10% buffered formalin and underwent tissue processing. To minimize bias, histological and immunohistochemical assessments (including cell counting and TNF-*α*/IL-6 expression scoring) were performed by a pathologist who was blinded to the experimental group allocations. For each section, five representative high-power fields (HPF) were examined at 400× magnification. Inflammatory cells (PMNs, lymphocytes, monocytes) and fibroblasts were identified by their morphological features and counted to obtain an average value per field. TNF-*α* and IL-6 expressions were identified by the presence of brown DAB chromogen and scored based on intensity and distribution. Statistical differences between groups were analyzed using One-Way ANOVA, with *p* < 0.05 considered statistically significant [95,96].

## 5. Conclusions

This study shows that the phytosome delivery system of the ethyl acetate fraction of *M. speciosa* fruit (PCFEAMS) has great potential as a topical antibacterial and anti-inflammatory agent. In vitro, this formula exhibits good physical stability with a nanoparticle size (244.60 ± 0.85 nm) and a high entrapment efficiency of 89%. In vivo testing indicated that PCFEAMS were able to reduce the number of *C. acnes* bacterial colonies, suppress pro-inflammatory cytokine levels (TNF-*α* and IL-6), and accelerate the tissue healing phase compared to regular fraction creams.

Although these results are promising, conclusions regarding clinical effectiveness are still limited to the scope of laboratory studies with limited sample sizes of test animals. Therefore, further research is urgently needed to validate these findings through acute irritation assays and long-term stability testing as per ICH guidelines, as well as clinical trials in humans to ensure safety and efficacy in acne vulgaris patients. The development of the formulation into lighter forms of preparations such as gels or serums is also recommended to improve the comfort of use on sensitive skin.

## Figures and Tables

**Table 1:** Antibacterial activity of ethanol extract, ethyl acetate fraction, *n*-hexane fraction, and water fraction at various concentrations against *C. acnes*.

Samples	Inhibitory Diameter (mm)			
	6.25 (mg/mL)	12.5 (mg/mL)	25.00 (mg/mL)	50.00 (mg/mL)
Ethanol Extract	9.50 ± 0.95	10.29 ± 0.84	13.34 ± 0.84 ****	15.40 ± 0.64 ****
Ethyl Acetate Fraction	11.67 ± 0.40 ****	14.79 ± 0.23 ****	15.83 ± 0.74 ****	17.49 ± 0.53 ****
*n*-hexane Fraction	7.73 ± 0.15	8.83 ± 0.51	9.39 ± 0.20	12.83 ± 0.80
Water Fraction	10.38 ± 0.58	10.75 ± 0.70 ****	11.72 ± 0.43 ****	14.36 ± 0.40 ****
Tetracycline 30 μg/mL	18.70 ± 0.96			
DMSO	0			

Inhibition zones are presented as mean ± SD from triplicate experiments (*n* = 3). Statistical significance was analyzed using One-Way ANOVA (*p* < 0.0001), followed by Tukey’s post hoc test for multiple comparisons. Asterisks (****) in the same row/column indicate statistically significant differences (*p* < 0.05).

**Table 2:** Characterization of phytosomes from *M. speciosa* Blume extract and EAFMS.

Formulas	Ratio (Extract/Fraction to Phospholipid)	Reflux Temp (°C)	Reflux Time (Hours)	Particle Size (nm)	Zeta Potential (mV)
Extract-Phytosome	1:3	50	3	835.96 ± 122.63	0.9 ± 0.45
EAFMS-Phytosome	1:3	50	3	256.70 ± 1.10	−82.7 ± 1.37

**Table 3:** Characterization of particle size, polydispersity index, zeta potential and entrapment efficiency at 25 °C and 40 °C over three months.

Formula	Characterization	Temp						
		25 °C				40 °C		
		0 Day	30 Days	60 Days	90 Days	30 Days	60 Days	90 Days
EAFMS-Phytosome	Particle Size (nm)	244.60 ± 0.85	249.10 ± 1.90	250.60 ± 1.41	252.70 ± 5.40	251.70 ± 4.35	252.70 ± 4.87	256.13 ± 1.92
	Polydispersity Index (PDI)	0.396 ± 0.08	0.529 ± 0.13	0.417 ± 0.14	0.450 ± 0.13	0.371 ± 0.02	0.53 ± 0.18	0.53 ± 0.13
	Zeta Potential (mV)	−56.70 ± 2.08	−57.40 ± 0.81	−53.56 ± 1.01	−53.36 ± 1.33	−58.26 ± 1.62	−53.90 ± 3.83	−54.70 ± 1.30
	*EE* (%)	89.46 ± 0.45	89.12 ± 0.44	89.12 ± 0.26	89.39 ± 0.51	89.49 ± 0.34	89.22 ± 0.18	89.39 ± 0.44

## Data Availability

The original contributions presented in this study are included in the article. Further inquiries can be directed to the corresponding author.

## References

[1] Deng Y., Wang F., He L. (2024). Skin Barrier Dysfunction in Acne Vulgaris: Pathogenesis and Therapeutic Approaches. Med. Sci. Monit..

[2] Vasam M., Korutla S., Bohara R.A. (2023). Acne Vulgaris: A Review of the Pathophysiology, Treatment, and Recent Nanotechnology Based Advances. Biochem. Biophys. Rep..

[3] Frediansyah A., Ramadaningrum W.A., Safitri I.O., Nabillah S.A.A., Aziz S.A.A., Utomo A.R.P. (2026). Phytochemical Composition, Health Benefits, and Potential Food Applications of Indonesian Native Parijoto (*Medinilla speciosa*) Fruits. Food Humanit..

[4] Wijayanti R., Wahyuono S., Puspitasari I., Rizal D.M. (2022). Isolation and Identification of Phytoconstituens from Methanol Extract Parijoto (*Medinilla speciosa*). Res. J. Pharm. Technol..

[5] Hu L., Luo Y., Yang J., Cheng C. (2025). Botanical Flavonoids: Efficacy, Absorption, Metabolism and Advanced Pharmaceutical Technology for Improving Bioavailability. Molecules.

[6] Berga M., Logviss K., Lauberte L., Paulausks A., Mohylyuk V. (2023). Flavonoids in the Spotlight: Bridging the Gap between Physicochemical Properties and Formulation Strategies. Pharmaceuticals.

[7] Parthasarathy S., Aly S.H., Tharumasivam S.V., Giridharan B., Chandran J., Thirumurthy P., Abd El Hafeez M.S., El-Shazly M. (2025). Unlocking Nature’s Secrets: A Review on the Pharmacokinetics of Plant-Based Medicines and Herbal Remedies. Nat. Prod. Res..

[8] Lee D., Kim J., Cong R., Park J., Nguyen C.H.B., Park K., Kang K., Shim S. (2025). Exploring Absorption Indices for a Variety of Polyphenols through Caco-2 Cell Model: Insights from Permeability Studies and Principal Component Analysis. J. Sci. Food Agric..

[9] Baradaran S., Hajizadeh Moghaddam A., Khanjani Jelodar S., Moradi-kor N. (2020). Protective Effects of Curcumin and Its Nano-Phytosome on Carrageenan-Induced Inflammation in Mice Model: Behavioral and Biochemical Responses. J. Inflamm. Res..

[10] Barani M., Sangiovanni E., Angarano M., Rajizadeh M.A., Mehrabani M., Piazza S., Gangadharappa H.V., Pardakhty A., Mehrbani M., Dell’Agli M. (2021). Phytosomes as Innovative Delivery Systems for Phytochemicals: A Comprehensive Review of Literature. Int. J. Nanomed..

[11] Albash R., Badawi N.M., Hamed M.I.A., Ragaie M.H., Mohammed S.S., Elbesh R.M., Darwish K.M., Lashkar M.O., Elhady S.S., Mosallam S. (2023). Exploring the Synergistic Effect of Bergamot Essential Oil with Spironolactone Loaded Nano-Phytosomes for Treatment of Acne Vulgaris: In Vitro Optimization, In Silico Studies, and Clinical Evaluation. Pharmaceuticals.

[12] Talebi M., Shahbazi K., Dakkali M.S., Akbari M., Almasi Ghale R., Hashemi S., Sashourpour M., Mojab F., Aminzadeh S. (2025). Phytosomes: A Promising Nanocarrier System for Enhanced Bioavailability and Therapeutic Efficacy of Herbal Products. Phytomed. Plus.

[13] Gupta M.K., Sansare V., Shrivastava B., Jadhav S., Gurav P. (2022). Comprehensive Review on Use of Phospholipid Based Vesicles for Phytoactive Delivery. J. Liposome Res..

[14] Li D., Zhou Z., Yang X., Zhang Q., Xu J., Zouboulis C., Xiang Q., Zhang S. (2025). A Comprehensive Review: The Bidirectional Role of Sebum in Skin Health. Bioengineering.

[15] Ameri A., Khazaeli P., Behnam B., Mehrabani M., Forootanfar H. (2024). Formulation and Optimization of Phytosomes of Ethanolic Extract of Viola Tricolor Flowers Using Design of Experiment (DOE) to Evaluate in Vitro Photoprotective Potential as Sunscreen Cream. Ind. Crops Prod..

[16] Freag M.S., Saleh W.M., Abdallah O.Y. (2018). Self-Assembled Phospholipid-Based Phytosomal Nanocarriers as Promising Platforms for Improving Oral Bioavailability of the Anticancer Celastrol. Int. J. Pharm..

[17] Chi C., Zhang C., Liu Y., Nie H., Zhou J., Ding Y. (2020). Phytosome-Nanosuspensions for Silybin-Phospholipid Complex with Increased Bioavailability and Hepatoprotection Efficacy. Eur. J. Pharm. Sci..

[18] Olfati A., Karimi N., Arkan E., Zhaleh M., Mozafari M.R. (2025). Enhancing Bioavailability and Stability of Plant Secondary Metabolites: Formulation and Characterization of Nanophytosomes Encapsulating Red Bryony and Horned Poppy Extracts. J. Funct. Biomater..

[19] Hendawy O.M., Al-Sanea M.M., Elbargisy R.M., Rahman H.U., Gomaa H.A.M., Mohamed A.A.B., Ibrahim M.F., Kassem A.M., Elmowafy M. (2023). Development of Olive Oil Containing Phytosomal Nanocomplex for Improving Skin Delivery of Quercetin: Formulation Design Optimization, In Vitro and Ex Vivo Appraisals. Pharmaceutics.

[20] Sun J., Shigemi H., Tanaka Y., Yamauchi T., Ueda T., Iwasaki H. (2015). Tetracyclines Downregulate the Production of LPS-Induced Cytokines and Chemokines in THP-1 Cells via ERK, P38, and Nuclear Factor-ΚB Signaling Pathways. Biochem. Biophys. Rep..

[21] Altayb H.N., Yassin N.F., Hosawi S., Kazmi I. (2022). In-Vitro and in-Silico Antibacterial Activity of Azadirachta Indica (Neem), Methanolic Extract, and Identification of Beta.d-Mannofuranoside as a Promising Antibacterial Agent. BMC Plant Biol..

[22] Sugiarti L., Fitrianingsih S. (2018). Aktivitas Antibakteri Ekstrak Etanol Daun Parijoto (*Medinilla Speciosa* Blume) Terhadap Pertumbuhan Bakteri Propionibacterium Acnes Dan Staphylococcus Aureus. Cendekia J. Pharm..

[23] Danaei M., Dehghankhold M., Ataei S., Hasanzadeh Davarani F., Javanmard R., Dokhani A., Khorasani S., Mozafari M.R. (2018). Impact of Particle Size and Polydispersity Index on the Clinical Applications of Lipidic Nanocarrier Systems. Pharmaceutics.

[24] Peanparkdee M., Yooying R. (2023). Enhancement of Solubility, Thermal Stability and Bioaccessibility of Vitexin Using Phosphatidylcholine-Based Phytosome. NFS J..

[25] Singh Y., Meher J.G., Raval K., Khan F.A., Chaurasia M., Jain N.K., Chourasia M.K. (2017). Nanoemulsion: Concepts, Development and Applications in Drug Delivery. J. Control. Release.

[26] Ruiz E., Orozco V.H., Hoyos L.M., Giraldo L.F. (2022). Study of Sonication Parameters on PLA Nanoparticles Preparation by Simple Emulsion-Evaporation Solvent Technique. Eur. Polym. J..

[27] Ribeiro A.M., Estevinho B.N., Rocha F. (2021). The Progress and Application of Vitamin E Encapsulation—A Review. Food Hydrocoll..

[28] Potdar S.B., Landge V.K., Barkade S.S., Potoroko I., Sonawane S.H. (2020). Flavor Encapsulation and Release Studies in Food. Encapsulation of Active Molecules and Their Delivery System.

[29] Demir B., Barlas F.B., Guler E., Gumus P.Z., Can M., Yavuz M., Coskunol H., Timur S. (2014). Gold Nanoparticle Loaded Phytosomal Systems: Synthesis, Characterization and in Vitro Investigations. RSC Adv..

[30] Németh Z., Csóka I., Semnani Jazani R., Sipos B., Haspel H., Kozma G., Kónya Z., Dobó D.G. (2022). Quality by Design-Driven Zeta Potential Optimisation Study of Liposomes with Charge Imparting Membrane Additives. Pharmaceutics.

[31] Lv Y., He H., Qi J., Lu Y., Zhao W., Dong X., Wu W. (2018). Visual Validation of the Measurement of Entrapment Efficiency of Drug Nanocarriers. Int. J. Pharm..

[32] Jacob S., Kather F.S., Boddu S.H.S., Rao R., Nair A.B. (2025). Vesicular Carriers for Phytochemical Delivery: A Comprehensive Review of Techniques and Applications. Pharmaceutics.

[33] Halevas E.G., Avgoulas D.I., Katsipis G., Pantazaki A.A. (2022). Flavonoid-Liposomes Formulations: Physico-Chemical Characteristics, Biological Activities and Therapeutic Applications. Eur. J. Med. Chem. Rep..

[34] Kalaivani P., Kamaraj R. (2024). Phytosome Technology: A Novel Breakthrough for the Health Challenges. Cureus.

[35] Drescher S., van Hoogevest P. (2020). The Phospholipid Research Center: Current Research in Phospholipids and Their Use in Drug Delivery. Pharmaceutics.

[36] Dashti A., Karamibonari A.R., Farahpour M.R., Tabatabaei Z.G. (2024). Topical Effectiveness of Eugenol Phytosome/Chitosome Hydrogels on the Healing Process of Infected Excision Wounds. Colloids Surf. A Physicochem. Eng. Asp..

[37] Bot F., Cossuta D., O’Mahony J.A. (2021). Inter-Relationships between Composition, Physicochemical Properties and Functionality of Lecithin Ingredients. Trends Food Sci. Technol..

[38] Xiang Y., Xiang M., Mao Y., Huang L., He Q., Dong Y. (2025). Insights into Structure-Antioxidant Activity Relationships of Polyphenol-Phospholipid Complexes: The Effect of Hydrogen Bonds Formed by Phenolic Hydroxyl Groups. Food Chem..

[39] Tafish A.M., El-Sherbiny M., Al-Karmalawy A.A., Soliman O.A.E.-A., Saleh N.M. (2023). Carvacrol-Loaded Phytosomes for Enhanced Wound Healing: Molecular Docking, Formulation, DoE-Aided Optimization, and In Vitro/In Vivo Evaluation. Int. J. Nanomed..

[40] Kumar M., Sharma A., Mahmood S., Thakur A., Mirza M.A., Bhatia A. (2024). Franz Diffusion Cell and Its Implication in Skin Permeation Studies. J. Dispers. Sci. Technol..

[41] Czajkowska-Kośnik A., Szekalska M., Winnicka K. (2019). Nanostructured Lipid Carriers: A Potential Use for Skin Drug Delivery Systems. Pharmacol. Rep..

[42] Awlqadr F.H., Majeed K.R., Altemimi A.B., Hassan A.M., Qadir S.A., Saeed M.N., Faraj A.M., Salih T.H., Abd Al-Manhel A.J., Najm M.A.A. (2025). Nanotechnology-Based Herbal Medicine: Preparation, Synthesis, and Applications in Food and Medicine. J. Agric. Food Res..

[43] Zouboulis C.C., Coenye T., He L., Kabashima K., Kobayashi T., Niemann C., Nomura T., Oláh A., Picardo M., Quist S.R. (2022). Sebaceous Immunobiology—Skin Homeostasis, Pathophysiology, Coordination of Innate Immunity and Inflammatory Response and Disease Associations. Front. Immunol..

[44] Mayslich C., Grange P.A., Dupin N. (2021). Cutibacterium Acnes as an Opportunistic Pathogen: An Update of Its Virulence-Associated Factors. Microorganisms.

[45] Thoraval L., Varin-Simon J., Ohl X., Velard F., Reffuveille F., Tang-Fichaux M. (2025). Cutibacterium Acnes and Its Complex Host Interaction in Prosthetic Joint Infection: Current Insights and Future Directions. Res. Microbiol..

[46] Talebi M., Bozorgchami N., Almasi Ghale R., Esmaeeli H., Moosavizadeh A., Aghajani A., Far B.F., Aminzadeh S. (2024). The Emerging Applications of Niosome as a Nanotechnology-Based Approach in Vaccine Delivery. Vacunas.

[47] Khan A.D., Singh M.K., Lavhale P.M., Yasir M., Singh L. (2024). Exploring the Wound Healing Activity of Phytosomal Gel of *Annona squamosa* and *Cinnamomum tamala* Leaves Ethanolic Extracts with Antioxidant and Antimicrobial Activities in *S Aureus* Infected Excision Wound Model. J. Biomater. Sci. Polym. Ed..

[48] Deleanu M., Toma L., Sanda G.M., Barbălată T., Niculescu L.Ş., Sima A.V., Deleanu C., Săcărescu L., Suciu A., Alexandru G. (2023). Formulation of Phytosomes with Extracts of Ginger Rhizomes and Rosehips with Improved Bioavailability, Antioxidant and Anti-Inflammatory Effects In Vivo. Pharmaceutics.

[49] Zulhendri F., Lesmana R., Tandean S., Christoper A., Chandrasekaran K., Irsyam I., Suwantika A.A., Abdulah R., Wathoni N. (2022). Recent Update on the Anti-Inflammatory Activities of Propolis. Molecules.

[50] Manfredini M., Bettoli V., Sacripanti G., Farnetani F., Bigi L., Puviani M., Corazza M., Pellacani G. (2019). The Evolution of Healthy Skin to Acne Lesions: A Longitudinal, In Vivo Evaluation with Reflectance Confocal Microscopy and Optical Coherence Tomography. J. Eur. Acad. Dermatol. Venereol..

[51] Feng Y., Li J., Mo X., Ju Q. (2024). Macrophages in Acne Vulgaris: Mediating Phagocytosis, Inflammation, Scar Formation, and Therapeutic Implications. Front. Immunol..

[52] Dahlan N.H., Sitohang I.B., Indriatmi W., Wibowo H., Enggy L.E. (2024). Correlation Between Reduced IL-1*β* Levels in Acne Lesions and the Decrease in Acne Inflammatory Lesions Following Topical Vitamin D Administration: A Double-Blind Randomized Controlled Trial. Clin. Cosmet. Investig. Dermatol..

[53] Griffin M.O., Fricovsky E., Ceballos G., Villarreal F. (2010). Tetracyclines: A Pleitropic Family of Compounds with Promising Therapeutic Properties. Review of the literature. Am. J. Physiol.-Cell Physiol..

[54] Orylska-Ratynska M., Placek W., Owczarczyk-Saczonek A. (2022). Tetracyclines—An Important Therapeutic Tool for Dermatologists. Int. J. Environ. Res. Public Health.

[55] Griffin M.O., Ceballos G., Villarreal F.J. (2011). Tetracycline Compounds with Non-Antimicrobial Organ Protective Properties: Possible Mechanisms of Action. Pharmacol. Res..

[56] Li Y., Hu X., Dong G., Wang X., Liu T. (2024). Acne Treatment: Research Progress and New Perspectives. Front. Med..

[57] Szlachcikowska D., Mazurek K., Magiera M., Jama G., Tabęcka-Łonczyńska A. (2025). Current Insights and Future Directions in Scar Management and Skin Regeneration. Int. J. Mol. Sci..

[58] Soliman A.M., Barreda D.R. (2022). Acute Inflammation in Tissue Healing. Int. J. Mol. Sci..

[59] Huang L., Yang S., Yu X., Fang F., Zhu L., Wang L., Zhang X., Yang C., Qian Q., Zhu T. (2024). Association of Different Cell Types and Inflammation in Early Acne Vulgaris. Front. Immunol..

[60] Yu Y., Shen Y., Zhang S., Wang N., Luo L., Zhu X., Xu X., Cong W., Jin L., Zhu Z. (2022). Suppression of Cutibacterium Acnes-Mediated Inflammatory Reactions by Fibroblast Growth Factor 21 in Skin. Int. J. Mol. Sci..

[61] Sánchez-Pellicer P., Navarro-Moratalla L., Núñez-Delegido E., Ruzafa-Costas B., Agüera-Santos J., Navarro-López V. (2022). Acne, Microbiome, and Probiotics: The Gut–Skin Axis. Microorganisms.

[62] Makrantonaki E., Ganceviciene R., Zouboulis C.C. (2011). An Update on the Role of the Sebaceous Gland in the Pathogenesis of Acne. Dermato-Endocrinology.

[63] Mayslich C., Grange P.A., Castela M., Marcelin A.G., Calvez V., Dupin N. (2022). Characterization of a Cutibacterium Acnes Camp Factor 1-Related Peptide as a New TLR-2 Modulator in In Vitro and Ex Vivo Models of Inflammation. Int. J. Mol. Sci..

[64] Soares C.L.R., Wilairatana P., Silva L.R., Moreira P.S., Vilar Barbosa N.M.M., da Silva P.R., Coutinho H.D.M., de Menezes I.R.A., Felipe C.F.B. (2023). Biochemical Aspects of the Inflammatory Process: A Narrative Review. Biomed. Pharmacother..

[65] Jin Z., Song Y., He L. (2023). A Review of Skin Immune Processes in Acne. Front. Immunol..

[66] Kim Y.-S., Kim H.S. (2024). Tetracyclines Revisited: Tetracyclines in the Field of Dermatology. Dermatology.

[67] Qi W., Wang R., Khasawneh S.M.S., Hui F., Liu Y. (2025). Levels of Several Inflammatory Cytokines in Acne Patients before and after Isotretinoin Therapy: A Randomized, Controlled Clinical Trial. J. Dermatol. Treat..

[68] Lu M., Qiu Q., Luo X., Liu X., Sun J., Wang C., Lin X., Deng Y., Song Y. (2019). Phyto-Phospholipid Complexes (Phytosomes): A Novel Strategy to Improve the Bioavailability of Active Constituents. Asian J. Pharm. Sci..

[69] Alharbi W.S., Almughem F.A., Almehmady A.M., Jarallah S.J., Alsharif W.K., Alzahrani N.M., Alshehri A.A. (2021). Phytosomes as an Emerging Nanotechnology Platform for the Topical Delivery of Bioactive Phytochemicals. Pharmaceutics.

[70] Koppula S., Shaik B., Maddi S. (2025). Phytosomes as a New Frontier and Emerging Nanotechnology Platform for Phytopharmaceuticals: Therapeutic and Clinical Applications. Phytother. Res..

[71] Pocino K., Carnazzo V., Stefanile A., Basile V., Guerriero C., Marino M., Rigante D., Basile U. (2024). Tumor Necrosis Factor-Alpha: Ally and Enemy in Protean Cutaneous Sceneries. Int. J. Mol. Sci..

[72] Hernández-Rosas N.A., Rivera-Yañez C.R., Hernández-Sánchez K.M., Nieto-Yañez O., Barrera-Ortega C.C., Gutiérrez-Rebolledo G.A., Pérez-Pastén-Borja R., Rivera-Yañez N., Ruiz-Hurtado P.A. (2025). Integrative Review of Flavonoid Phytosomes and Their Potential Role in the Treatment of SARS-CoV-2 Infection (COVID-19), Diabetes, and Cancer. Appl. Food Res..

[73] Sandoval A.G.W., Vaughn L.T., Huang J.T., Barbieri J.S. (2023). Role of Tumor Necrosis Factor–α Inhibitors in the Treatment and Occurrence of Acne. JAMA Dermatol..

[74] Younis S., Shamim S., Nisar K., Deeba F., Mehmood S., Mumtaz S., Blumenberg M., Javed Q. (2021). Association of TNF-*α* Polymorphisms (−857, −863 and −1031), TNF-*α* Serum Level and Lipid Profile with Acne Vulgaris. Saudi J. Biol. Sci..

[75] Johnson B.Z., Stevenson A.W., Prêle C.M., Fear M.W., Wood F.M. (2020). The Role of IL-6 in Skin Fibrosis and Cutaneous Wound Healing. Biomedicines.

[76] Thukham-Mee W., Wattanathorn J., Palachai N. (2025). SIRT1-Mediated Epigenetic Protective Mechanisms of Phytosome-Encapsulated Zea Mays L. Var. Ceratina Tassel Extract in a Rat Model of PM2.5-Induced Cardiovascular Inflammation. Int. J. Mol. Sci..

[77] Aggarwal D., Chaudhary M., Mandotra S.K., Tuli H.S., Chauhan R., Joshi N.C., Kaur D., Dufossé L., Chauhan A. (2025). Anti-Inflammatory Potential of Quercetin: From Chemistry and Mechanistic Insight to Nanoformulations. Curr. Res. Pharmacol. Drug Discov..

[78] Saini K., Modgill N., Singh K., Kakkar V. (2022). Tetrahydrocurcumin Lipid Nanoparticle Based Gel Promotes Penetration into Deeper Skin Layers and Alleviates Atopic Dermatitis in 2,4-Dinitrochlorobenzene (DNCB) Mouse Model. Nanomaterials.

[79] Marques M.P., Varela C., Mendonça L., Cabral C. (2023). Nanotechnology-Based Topical Delivery of Natural Products for the Management of Atopic Dermatitis. Pharmaceutics.

[80] Dai F., Cao J., Liu N., Peng M., Wang C. (2024). Determination and Correlation of LLE Data for N-Hexane, Ethyl Acetate and Different Extractants. J. Chem. Thermodyn..

[81] Mardiana L., Milanda T., Hadisaputri Y.E., Chaerunisaa A.Y., Puspadewi R. (2026). Anticancer Mechanisms of Poikilospermum Suaveolens Root Fraction: Ethnopharmacological Relevance, Signaling Pathways, and in Vitro/in Silico Studies on MCF-7 Cells. J. Ethnopharmacol..

[82] Chang C.-C., Yang M.-H., Wen H.-M., Chern J.-C. (2020). Estimation of Total Flavonoid Content in Propolis by Two Complementary Colometric Methods. J. Food Drug Anal..

[83] Baba S.A., Malik S.A. (2015). Determination of Total Phenolic and Flavonoid Content, Antimicrobial and Antioxidant Activity of a Root Extract of Arisaema Jacquemontii Blume. J. Taibah Univ. Sci..

[84] AlSedairy S.A., Binobead M.A., Alanazi F., Aziz I.M. (2026). Evaluation of the Antibacterial, Antioxidant, Anticancer, and Antidiabetic Activities of the Leaves and Inflorescences of Crassula Capitella. Biomedicines.

[85] Chaerunisaa A.Y., Dewi M.K., Sriwidodo J.I., Joni I.M., Dwiyana R.F. (2022). Development of Cathelicidin in Liposome Carrier Using Thin Layer Hydration Method. Int. J. Appl. Pharm..

[86] Sahibzada M.U.K., Sadiq A., Khan S., Faidah H.S., Ullah N., Khurram M., Amin M.U., Haseeb A. (2017). Fabrication, Characterization and in Vitro Evaluation of Silibinin Nanoparticles: An Attempt to Enhance Its Oral Bioavailability. Drug Des. Devel. Ther..

[87] Brodin B., Steffansen B., Nielsen C.U. (2010). Passive Diffusion of Drug Substances: The Concepts of Flux and Permeability.

[88] Pulsoni I., Lubda M., Aiello M., Fedi A., Marzagalli M., von Hagen J., Scaglione S. (2022). Comparison Between Franz Diffusion Cell and a Novel Micro-Physiological System for In Vitro Penetration Assay Using Different Skin Models. SLAS Technol..

[89] (2003). Stability Testing of New Drug Substances and Products Step 5 Note for Guidance on Stability Testing of New Drug Substances and Products.

[90] Taleuzzaman M., Sartaj A., Kumar Gupta D., Gilani S.J., Mirza M.A. (2023). Phytosomal Gel of Manjistha Extract (MJE) Formulated and Optimized with Central Composite Design of Quality by Design (QbD). J. Dispers. Sci. Technol..

[91] Jain P., Taleuzzaman M., Kala C., Kumar Gupta D., Ali A., Aslam M. (2021). Quality by Design (Qbd) Assisted Development of Phytosomal Gel of Aloe Vera Extract for Topical Delivery. J. Liposome Res..

[92] Machmudah S., Trisanti P.N., Widiyastuti, Wahyudiono, Adschiri T., Goto M. (2024). Liposomal Encapsulation of Curcumin Employing Soy Lecithin in Ultrasonic Environment under Dense Carbon Dioxide. Alex. Eng. J..

[93] Pornpattananangkul D., Fu V., Thamphiwatana S., Zhang L., Chen M., Vecchio J., Gao W., Huang C., Zhang L. (2013). In Vivo Treatment of Propionibacterium Acnes Infection with Liposomal Lauric Acids. Adv. Healthc. Mater..

[94] Ren X., Zhou N., Li D., Li L., Wang Y., Li L., Ma Y., Gao X., Zhao Y., Sun Y. (2025). Network Pharmacology, Transcriptomics, and Biological Validation Reveal a Lipid Secretion Inhibitory and Anti-Inflammatory Mechanism of Tanreqing Gel in the Treatment of Acne. J. Ethnopharmacol..

[95] Varghese F., Bukhari A.B., Malhotra R., De A. (2014). IHC Profiler: An Open Source Plugin for the Quantitative Evaluation and Automated Scoring of Immunohistochemistry Images of Human Tissue Samples. PLoS ONE.

[96] Fedchenko N., Reifenrath J. (2014). Different Approaches for Interpretation and Reporting of Immunohistochemistry Analysis Results in the Bone Tissue—A Review. Diagn. Pathol..

